# **Responses of intestinal organoids to infection by** ***Mycobacterium avium*** **resemble symptoms observed in Crohn’s disease**

**DOI:** 10.1080/19490976.2026.2630483

**Published:** 2026-02-13

**Authors:** Wanbin Hu, Adriana Martinez Silgado, Ninouk Akkerman, Ronald W.A.L. Limpens, Roman I. Koning, Hans Clevers, Herman P. Spaink

**Affiliations:** a Institute of Biology Leiden, Leiden University, Leiden, The Netherlands; b Oncode Institute, Hubrecht Institute, Royal NetherlandsAcademy of Arts and Sciences (KNAW) and UMC Utrecht, Utrecht, The Netherlands; c Electron Microscopy Facility, Department of Cell and Chemical Biology, Leiden University Medical Center, Leiden, Netherlands

**Keywords:** *Mycobacterium avium*, Crohn’s disease, small intestinal organoids, MMP7, serial block-face scanning electron microscopy

## Abstract

Crohn’s disease (CD) is a chronic inflammatory bowel disease (IBD). *Mycobacterium avium*, which causes Johne’s disease in ruminants, has been suggested as a potential CD trigger due to shared pathology, but early epithelial responses remain unclear. This study established a mouse small intestinal organoid (mSIO) model of *M. avium* infection to assess CD-related inflammation. Infected mSIOs were examined by confocal microscopy, block-face scanning electron microscopy, and macrophage co-culture to track bacterial localization and immune cell behavior. The data give unprecedent dynamic and super high resolution insights in the responses of gut cells to mycobacterial infection. RNA-seq with GSEA revealed strong induction of inflammatory genes and enrichment of pro-inflammatory pathways. Comparative analysis with CD-humanized mouse data showed overlapping gene expression and enrichment of the IBD signaling pathway. Notably, *Mmp7*, which can be linked to epithelial remodeling and inflammation, was a common marker in both models. This study presents a robust mSIO model of *M. avium* infection that recapitulates features of CD-associated inflammation both with high-resolution imaging and transcriptomics and identifies *Mmp7* as a potential molecular link between infection and CD-like pathology.

## Introduction

Crohn’s disease (CD) is a chronic and relapsing form of inflammatory bowel disease (IBD) characterized by transmural intestinal inflammation, disruption of epithelial integrity, and dysregulated immune responses.^1^ Although the etiology of CD remains multifactorial, there are indications that suggest a central role for host–microbe interactions in disease onset and progression.^2^ In particular, intestinal dysbiosis and aberrant immune responses to commensal or pathogenic microbes contribute to the chronic inflammation observed in patients.

Many bacterial strains belonging to the *Mycobacterium avium complex* (MAC) are notorious as intestinal pathogens in a large variety of animals. For instance, *M. avium subsp. paratuberculosis* (MAP) causes a chronic wasting syndrome and colitis symptoms in ruminants called Johne’s disease (JD).^3^ Although CD is more linked to host-microbe interactions and dysregulated immune responses, a growing number of studies suggest that there is a strong correlation of the presence of *M. avium* bacteria in intestinal lesions or blood of many other animal species and humans with IBD symptoms.^4^^-^^6^ However, it is still heavily debated whether *M. avium* bacteria are causative agents of IBD, such as CD, in human patients.^7^^-^^9^ This is particularly hard to demonstrate because of the histological similarity of the intestinal lesions found in CD and intestinal tuberculosis.^10^ Recent studies have therefore aimed at obtaining markers to discriminate between these diseases in human patient material.^11^ However, at the cellular and molecular levels little is still known about the mechanisms of infection of the intestines by mycobacteria, partly due to the lack of physiologically relevant models.

Organoids are 3D cultures from stem or primary cells that self-organize into organ-like structures with appropriate growth factors and hydrogels.^12^ Intestinal organoids offer greater cellular diversity and physiological relevance than 2D cultures, making them valuable for gut disease modeling.^13^ The development of long-term culture systems for intestinal organoids, first established by Sato et al., has provided a powerful model to study intestinal biology and host-pathogen interactions.^14^ Moreover, Pleguezuelos-Manzano et al. demonstrated that long-term exposure of intestinal organoids to colibactin-producing *E. coli* can induce a distinct mutational signature linked to colorectal cancer.^15^ Some studies have utilized patient-derived intestinal organoids to investigate the genetic features associated with CD.^16^^-^^18^ Additionally, bovine intestine-derived organoids have been developed and used to study MAP infection in ruminants.^19^ Despite these advances, the mechanisms underlying intestinal infection by *M. avium*—particularly with regard to epithelial responses—remain largely unknown. In particular, the early host epithelial responses to *M. avium* infection and their potential relevance to CD pathogenesis have not been systematically investigated. Thus, an organoid-based *M. avium* infection model may offer valuable insight into these early host–pathogen interactions.

In this study, we established a mouse small intestinal organoid (mSIO) model of *M. avium* infection to investigate its immunological and transcriptional impact, and to evaluate its potential relevance to CD-associated inflammation. Utilizing a combination of imaging, transcriptomic profiling, and comparative analysis with a published humanized mouse model of CD,^20^ we investigated the extent to which *M. avium-*induced inflammation in mSIOs reflects the inflammatory signatures of CD. We also found conserved molecular markers, including *Mmp7*, which may play a role in epithelial remodeling and inflammation. Our findings provide new insights into mycobacteria-associated epithelial inflammation and establish an *ex vivo* model for further study of host-microbe interactions in IBD.

## Materials and methods

### Ethics statement for animals and human participants

No human participants or live animals were involved in this study. The mSIOs used in this study were expanded entirely *in vitro* from an established mSIOs line previously described by Krueger et al.,^21^ and no new animals were used for this study. Briefly, the mSIOs used in this study were originally derived from C57BL/6J mice (*Mus musculus*; RRID: IMSR_JAX:000664), male and female, 7–12 weeks old. The animals were housed and maintained by professional caretakers under institutional guidelines and according to protocols approved by the Central Committee Animal Experimentation (CCD) of the Dutch government and the KNAW/Hubrecht Institute Animal Welfare Body (IvD), with project license number AVD8010020151.

### Organoid maintenance

MSIOs were established and cultured as previously described.^22^ Briefly, mSIOs were cultured in basement membrane extract (BME; Biotechne R&D Systems) and ENR growth medium (Advanced DMEM/F12, Life Technologies) supplemented with100 ng/mL murine recombinant Noggin and 500 ng/mL human recombinant Rspondin1 (Peprotech), 1 mM *n*-acetylcysteine (Sigma-Aldrich), 50 ng/mL epidermal growth factor (EGF), 1 × B27 supplement, 2 mM Glutamax, 10 mM HEPES, 100 U/mL Penicillin, 100 μg/mL Streptomycin (Penicillin-Streptomycin, Gibco Thermo Fisher Scientific). Media was refreshed every 2 days and mSIOs were passaged once a week as previously described.^22^^,^^23^

### Bacterial strain culture and mSIO infection

The *M. avium* Chester (also called MAC 101, ATCC® 700898™) containing the Wasabi expression vector pSMT3 (plasmid 26589, Addgene)^24^ was used in this study to induce infections. *M. avium* MAC 101 stain was originally isolated from the blood of an AIDS patient with disseminated *M. avium* infection. *M. avium* were grown at 37◦C in Middlebrook 7H9 broth with acid-albumin-dextrose-catalase (ADC) enrichment or Middlebrook 7H10 agar with 10% oleic acid-albumin-dextrose-catalase (OADC) enrichment with hygromycin 50 µg/mL. Bacteria were prepared one day before infection by adjusting the culture to an optical density at 600 nm (OD₆₀₀) of 0.2–0.3. OD₆₀₀ was measured again on the day of infection, with an OD₆₀₀ value of 1.0 corresponding to approximately 10⁸ *M. avium*/mL.

**Figure 1.**
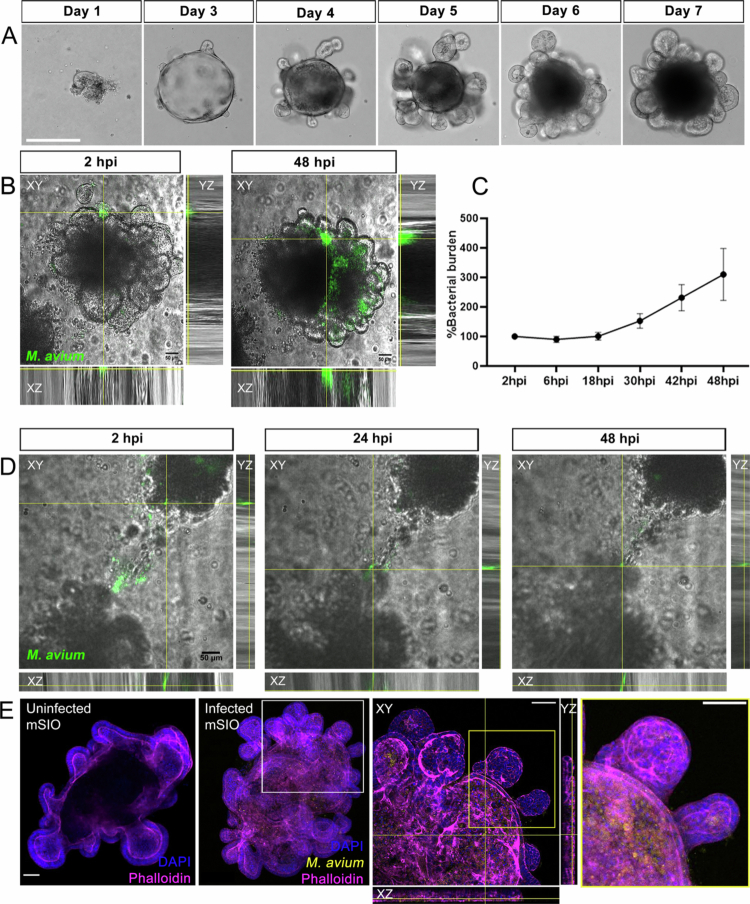
Establishment of a mouse small intestinal organoid model for *Mycobacterium avium* infection. (A) An example of a successfully cultured mouse small intestinal organoid (mSIO). Scale bar: 275 µm. (B) Representative images of mWasabi-expressing *M. avium* MAC101 infected-mSIO at 2 hour post infection (hpi) and 48 hpi. Scale bar: 50 µm. See Supplementary movie 1 and movie 2 to see the bacterial burden changing between 2 hpi and 48 hpi. (C) Quantification of normalized bacterial burden in the *M. avium*-infected mSIO from 2 hpi to 48 hpi. Fluorescent pixel counts of mWasabi-expressing *M. avium* were quantified, and bacterial burden at later time points was normalized to the fluorescent pixel count at 2 hpi (set to 1). Data were shown as mean ± SEM (*n* = 3). (D) Uninfected mSIOs migrate to *M. avium*. Scale bar: 50 µm. See Supplementary movie 3 for the living images of panel (D) and movie 4 for another example of uninfected mSIO migrating to *M. avium*. (E) Phalloidin staining of an uninfected mSIO and an *M.* *avium*-infected mSIO. mWasabi-expressing *M. avium* (yellow)-infected mSIO was fixed and stained at 24 hpi by phalloidin-Alexa568 (magenta). Nuclei were stained by DAPI (blue). Scale bar: 100 µm. See Supplementary movie 5 for the 3D reconstruction of the *M.* *avium*-infected mSIO. Scale bar: 100 µm.

MSIOs were released from BME using cold mENR medium after 7 days of mechanical splitting. To initiate infection, approximately 10 mSIOs were seeded into a glass-bottom 96-well plate (Greiner, 655892) together with 100,000 CFU of *M. avium* MAC101 expressing mWasabi. This dose was selected based on preliminary titration experiments (10⁴–10⁶ CFU), which showed that lower doses resulted in inconsistent colonization of day-7 mSIOs, whereas 10⁵ CFU produced reproducible infection without overt cytotoxicity. The interaction of *M. avium* and the mSIOs was observed under a confocal laser scanning microscope (CLSM) for 48 h at 37 °C 5% CO_2_. To further observe the interaction between *M. avium* and mSIOs, a robot injector (Life Science Methods, Netherlands) was used to directly inject *M. avium* into the BME Matrigel between two mSIOs (Figure 1D).

### Establishment and infection of macrophages and mSIOs co-culture

RAW 264.7 cell lines were cultured in DMEM high glucose medium supplemented with 10% FBS in the incubator with 5% CO_2_ and 95% humidity at 37 °C. RAW 264.7 cell lines were harvested after reaching 90% confluence (~1 × 10^6^/mL), and detached by using a cell scraper.^25^ RAW 264.7 macrophages were stained with 10 µM CellTracker^TM^ CM-DiI (C7000, Invitrogen) based on the previously published protocol.^26^

For co-culture experiments, approximately 100,000 CFU of *M. avium* MAC 101 were added to ~10 mSIOs and incubated at 37 °C for 20 minutes to allow initial infection. After this incubation, the infected mSIOs were mixed with ~10,000 macrophages and embedded in 70 µL of BME per well of a glass-bottom 96-well plate (655892, Greiner). The plate was incubated at 37 °C for 15 minutes to allow the BME to solidify, followed by the addition of 200 µL mENR medium to each well.

### TLR2 inhibitor treatment

To investigate the role of TLR2 in the mycobacteria-infected macrophages and mSIOs co-culture system, the TLR2 antagonists MMG-11 (HY-112146, MedChemExpress) and CU-CPT22 (HY-108471, MedChemExpress) were applied based on a previous study.^27^ Raw 264.7 macrophages (~10,000 cells/well) were seeded in a 96-well plate and washed with phosphate-buffered saline (PBS, Sigma Aldrich). MMG-11 and CU-CPT22 were made into 50 mM stock solutions and dissolved in DMSO. Subsequently, the cell culture media were changed to OptiMEM (Thermo Fisher Scientific, Darmstadt, Germany) for the inhibitor treatment. The cells were incubated with the MMG-11 (25 µM) or CU-CPT22 (25 µM) for 1 h before the co-culture.

### Live imaging and image analysis

To investigate the interaction between *M. avium* and mSIOs (Figure 1B, Figure 1D), as well as macrophage migration in the mSIO–macrophage co-culture system (Figure 3), live-cell imaging was performed using a Nikon Eclipse Ti-E microscope equipped with a Plan Apo 20 × /0.75 NA objective. Time-lapse imaging was conducted in a stage-top incubator maintained at 37 °C, with 5% CO₂ and 95% humidity, capturing images every 30 minutes for a total of 48 hours. The integrated fluorescence intensity of *M. avium* (Figure 1C) was quantified using ImageJ. For 3D reconstruction shown in Figure 1E, the Surface tool in Imaris software (version 10.2) was used. The cell tracking of macrophages was performed by using half- automatically tracking plug-in TrackMate.^28^

### Serial block-face scanning electron microscopy

Samples were prepared for serial block-face scanning electron microscopy (SBF-SEM) according to a slightly adapted protocol from Deerink et al.^29^ After fixing the material for 2 hours at room temperature with 2,5% Glutaraldehyde + 2% Paraformaldehyde in 0.15 M Cacodylate buffer containing 2 mM CaCl_2_, the material was washed 3 times with Cacodylate buffer and then placed into 2% OsO_4_/1.5% potassium ferrocyanide in 0.15 M Cacodylate buffer containing 2 mM CaCl_2_. The material was left for 60 minutes on ice. After washing 3 times in milliQ water, the material was placed into 1% Thiocarbohydrazide for 20 minutes at room temperature. The material was again washed with milliQ water and then stained with 2% aqueous OsO_4_ for 30 min at room temperature. After washing 3 times with milliQ water, the material was placed into 1% Uranyl acetate for 2 hours at room temperature. The material was washed with milliQ water then stained with Lead aspartate for 30 minutes at 60 °C. The material was washed with milliQ water and then dehydrated on ice in 20%, 50% and 70% ethanol solutions for 5 minutes at each step. After replacing the 70% ethanol with a fresh 70% ethanol solution, the samples were kept overnight at 4 °C. The next day, samples were dehydrated in 90%, 100%, 100% ethanol solutions for 5 minutes at each step. Next, the material was kept in dry acetone for 10 minutes on ice, and another 10 minutes in fresh dry acetone at room temperature. The material was infiltrated with 25%, 50% and 75% Spurr (Sigma-Aldrich) solution in acetone for 2 hours at room temperature each step, followed by an overnight step at room temperature in 100% Spurr resin (Sigma-Aldrich). The next day, the material was placed in fresh Spurr resin for 2 hours at room temperature, after which the material was embedded and polymerized at 60 °C for 48 hours.

Data was collected with a Gatan 3View2XP unit installed on a Zeiss Gemini 300 field emission SEM. From both uninfected and infected mSIOs three volumes were recorded containing between 68 (controls) to 1153 sections. Images were collected at 1.6 kV accelerating voltage and focal charge compensator at 65%. The pixel dwell time was 3 microseconds, the pixel sizes were 10, 15, 20, and 75 nm, while the section thickness was 100 nm. Volumes were annotated by hand using MIB^30^ and volume renderings were made using Amira 2024.2 (Thermo Fischer Scientific) and Cinema4D 24.1111 (Maxon).

### RNA isolation, RNA-seq and data analysis

To compare the difference between the uninfected and *M. avium*- infected mSIOs, we infected mSIOs with *M. avium* as described above. ~30 mSIOs per group were harvested for total RNA isolation using TRIzol Reagent (Life Technologies). Subsequently, genomic DNA contamination was removed through DNase treatment according to the manufacturer's instructions (Thermo Fisher Scientific). RNA quantity and purity were assessed using a NanoDrop 2000 spectrophotometer (Thermo Fisher Scientific).

RNA sequencing (RNA-seq) was performed on four biological replicates per group by BGI Genomics (Hong Kong, China) using the DNBSEQ platform. Sequencing reads were aligned to the *Mus musculus* reference genome (GRCm39) using CLC Genomics Workbench (Cat. 832583, QIAGEN). Across all samples, more than 90% of reads successfully mapped to the reference genome, indicating high alignment quality. Principal component analysis (PCA) was conducted using the RNA-seq PCA tool in CLC Genomics Workbench to assess sample clustering and variance. Differentially expressed genes (DEGs) were identified using the "Differential Expression in Two Groups" tool in CLC, applying a false discovery rate (FDR)-adjusted *p*-value threshold.

Gene Set Enrichment Analysis (GSEA) was performed using the C2 “Curated Gene Sets” collection from the Molecular Signatures Database (MSigDB), as previously described.^31^ To investigate whether *M. avium*-infected mSIOs exhibit CD-associated transcriptional features, we compared our dataset with a publicly available transcriptomic dataset derived from germ-free mice colonized with fecal microbiota from either healthy donors or CD patients (BioProject PRJNA980747).^20^

### qRT-PCR

To validate the RNA-seq results for *Mmp7* expression in *M. avium*-infected mSIOs, quantitative real-time PCR (qRT-PCR) was performed. Total RNA was extracted as described above, and 200 ng of RNA was reverse-transcribed into cDNA using the iScript™ cDNA Synthesis Kit (Bio-Rad). The forward primer sequence of *Mmp7* is 5’ GCAGACAGATCACAGAAACGG 3’ and the reverse primer sequence is 5' GCACAAGGAAGAGGGAAACA 3'. *β-actin* was used as a reference gene. The forward primer sequence of *β*-actin is 5’ GGCTATGCTCTCCCTCACG 3’, and the reverse primer sequence is 5' CACGCTCGGTCAGGATCTT 3’. qRT-PCR was conducted on a CFX96 Real-Time PCR Detection System (Bio-Rad) using iQ™ SYBR Green Supermix (Bio-Rad). Relative gene expression levels were calculated using the ΔΔCt method.

### Western blot

To assess MMP7 protein expression in *M. avium*-infected mSIOs, Western blot analysis was performed. MSIOs were lysed in RIPA buffer (Thermo Fisher Scientific) supplemented with protease and phosphatase inhibitors (Roche). Protein concentrations were determined using the BCA Protein Assay Kit (Thermo Fisher Scientific). Equal amounts of protein (typically 20–30 μg per lane) were separated by SDS-PAGE on a 10% polyacrylamide gel and transferred to a PVDF membrane (Millipore). Membranes were blocked with 5% non-fat milk in TBS-T (Tris-buffered saline with 0.1% Tween-20) for 1 hour at room temperature and incubated overnight at 4 °C with primary antibodies MMP7 (3801, Cell Signaling D4H5) and *β*-actin (4970, Cell Signaling 13E5) (loading control). After washing, membranes were incubated with HRP-conjugated secondary antibodies (Cell Signaling Technology) for 1 hour at room temperature. Protein bands were visualized using enhanced chemiluminescence (ECL) substrate (Bio-Rad), and images were acquired with a ChemiDoc Imaging System (Bio-Rad).

### Immunofluorescence staining and imaging

Immunofluorescent staining was performed to localize MMP7 expression and assess epithelial architecture in mSIOs. MSIOs were fixed in 4% paraformaldehyde for 30 minutes at room temperature, washed with PBS, and permeabilized with 0.3% Triton X-100 for 20 minutes. Samples were then blocked in 5% BSA in PBS for 1 hour at room temperature, followed by overnight incubation at 4 °C with a primary antibody against MMP7 (3801, Cell Signaling D4H5, 1:1000 dilution). After PBS washes, samples were incubated with Alexa Fluor-conjugated secondary antibodies (Thermo Fisher Scientific) for 1 hour at room temperature.

Subsequently, Phalloidin (for F-actin labeling) was diluted in 5% BSA (1:20) and applied to the samples for 4 hours at room temperature. After three PBS washes, nuclei were counterstained with DAPI. Finally, mSIOs were mounted using ProLong™ Gold Antifade Mountant (Invitrogen) and imaged with a Leica SP8 confocal laser scanning microscope.

## Statistical analysis

Statistical analyses were performed using GraphPad Prism software (version 10.1.2; San Diego, CA, USA). Data are presented as mean ± standard error of the mean. The Shapiro–Wilk test was used to assess the normality of data distribution. For normally distributed data, unpaired two-tailed t-tests were used to assess significance. A *P* value < 0.05 was considered statistically significant; *P* < 0.01 is indicated as **.

## Results

### Establishment of an mSIO infection model with *M. avium*

MSIOs were generated following the protocols of a previous publication.^22^ Figure 1A presents a representative example of successful mSIO culture, from the first day after passage (Day 1) to Day 7. To establish the infectious model, ~10 mSIOs were infected with ~100,000 CFU *M. avium* in a 96-well plate. The infected mSIOs were imaged from 2 hour post infection (hpi) to 48 hpi using CLSM. Representative images at 2 hpi and at 48 hpi are shown in Figure 1B (see Supplementary movies 1 and 2). At 24 hpi, approximately 75% of mSIOs were successfully infected by *M. avium* (Supplementary Figure 1). The normalized bacterial burden of the infected mSIOs increased progressively over time (Figure 1C). Unexpectedly, mSIOs basolateral side of cells exhibited directed movement toward *M. avium* microinjected directly into the BME Matrigel (Figure 1D; Supplementary movies 3 and 4). To localize the *M. avium* bacteria inside the mSIOs, phalloidin staining was performed. As shown in Figure 1E, both uninfected and *M. avium*-infected mSIOs at 24 hpi were examined. *M. avium*, fluorescently labeled with mWasabi protein, was detected within the cells of the infected mSIOs at 24 hpi (Figure 1E, Supplementary movie 5).

**Figure 2.**
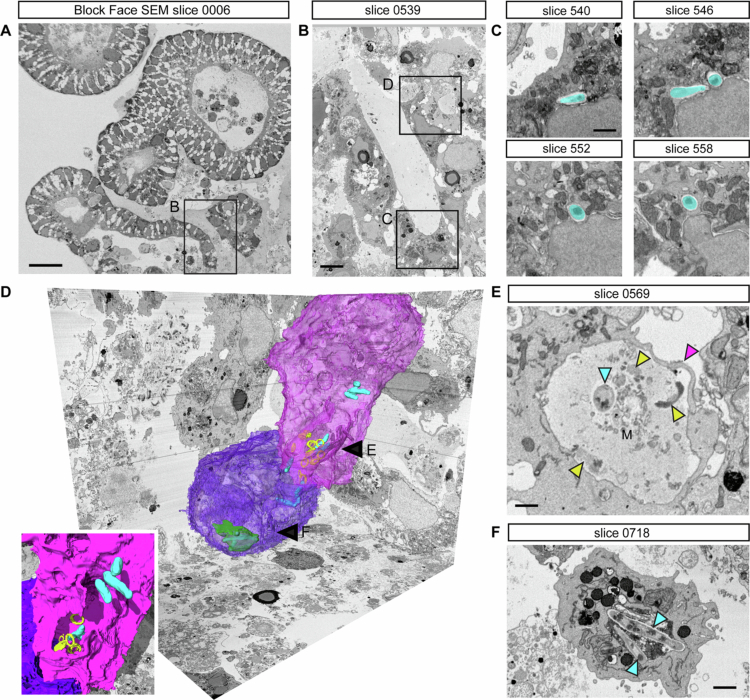
*M. avium* localizes intracellular within cells of mSIOs. (A) Serial block-face scanning electron microscopy (SBF-SEM) slice overview of *M. avium* infected mSIO and (B) zoomed image about 50 µm deeper in the block. (C) Four slices at 600 nm spacing through a cell containing two *M. avium* bacteria (cyan). See supplementary movie 6 for the 3D surface rendering of the *M. avium* bacteria inside the infected cells. (D) 3D surface renderings of two cells (purple and blue), engulfing *M. avium* (cyan) in a cavity containing ring-shaped degraded granule remnants (yellow, E). Inside the blue cell, *M. avium* are present inside what appears to be a lysosome/autophagosome (green, F). (E) Phagocytosis-like engulfment (magenta arrowheads) of the Matrigel (M) containing *M. avium* bacteria (cyan arrowhead), and remnants of exocytosed and partly degraded granules (yellow arrowheads). (F) View of *M. avium* inside the phagosome of the blue cell.

To validate these findings, SBF-SEM was employed (Figures 2A-C, Supplementary movie 6). Consistent with the confocal imaging results of phalloidin staining, *M. avium* was observed residing within cells (Figure 2C). These findings confirm that *M. avium* is capable of penetrating and infecting the interior of mSIOs following external exposure. In addition, SBF-SEM revealed a *M. avium* bacterium undergoing phagocytosis-like internalization by a cell from the basolateral side of the mSIO (Figure 2D-F), showing that cells in mSIOs may engage in phagocytosis-like uptake of *M. avium*, which is consistent with prior observations (Figure 1D). These findings show that cells in the mSIOs model are not only capable of sensing *M. avium* infection but may also exhibit primitive phagocytic behavior.

### *M. avium* infection drives macrophage recruitment toward the mSIOs

**Figure 3.**
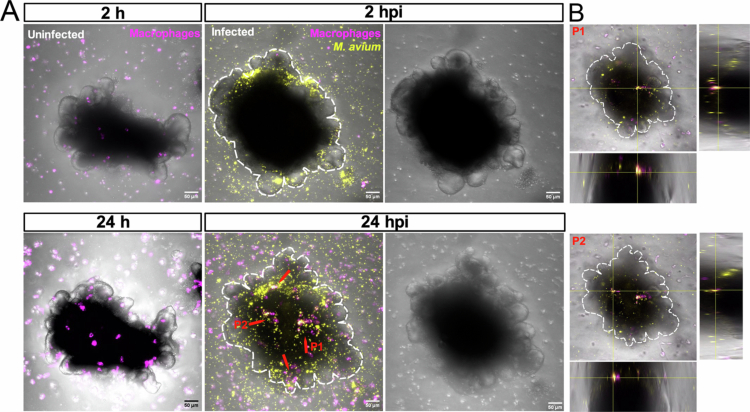
Macrophages migrate toward *M. avium*-infected mSIO. (A) Different time points following *M. avium* infection in a macrophage and mSIO co-culture system (yellow, *M. avium* MAC 101 wasabi; magenta, RAW 264.7 macrophages). Red arrows represent macrophages on the surface of the *M. avium*-infected mSIO. Scale bar: 50 µm. (B) Cross sections of *M. avium-*infected macrophages and mSIO from the z-stack images of the P1 and P2 from the panel (A). Scale bar: 50 µm. Representative image of an infected co-culture sample, selected from one of eight samples derived from three independently seeded wells, showing macrophage clustering around infected organoids. See Supplementary movie 7 for an example of macrophages migrating toward an *M. avium*-infected organoid.

Infected macrophages recruit additional macrophages and neutrophils, eventually leading to granuloma formation—inflammatory clusters that form the pathological hallmark of tuberculosis. Granuloma formation during mycobacterial infection has been shown to depend on the induction of matrix metalloproteinase-9 (MMP9) in epithelial cells neighboring infected macrophages leading to enhanced recruitment of macrophages.^32^ To recapitulate this aspect of pathogenesis in mSIOs model, we employed a co-culture system of mSIOs and macrophages, as previously described.^33^ Over time, macrophages were observed to migrate toward and cluster around the *M. avium*-infected mSIOs (Figure 3; Supplementary movie 7). The clustering migration pattern of macrophages around infected mSIOs, as shown in Figure 3, was consistently observed across eight co-culture samples derived from three independently seeded wells. In contrast, no such clustering was observed in uninfected mSIOs (Figure 3A) or in 3D cultures of RAW264.7 macrophages infected with *M. avium* (Supplementary Figure 2). Notably, infected macrophage clusters were found to infiltrate the mSIO by 24 hpi (Figure 3B). The coordinated responses between infected mSIOs and immune cells reflect early events in granuloma formation, highlighting the utility of this model for dissecting host-pathogen interactions at the epithelial-immune interface and further investigation of the early molecular responses of mSIOs to infection.

To determine the role of TLR2 in the behavior of macrophages towards infected mSIOs, we tested the effect of inhibitors of TLR2. TLR2 inhibitors MMG-11 and CU-CPT22 are well-characterized competitive antagonists of TLR2 heterodimers by competitively binding to the same site as Pam3CSK4 or Pam2CSK4.^27^ Macrophages were pre-treated with the TLR2 inhibitors or DMSO as previously described.^27^ Subsequently, inhibitor-treated macrophages were seeded with mSIOs as described above. Live imaging was performed to observe macrophage behavior (Supplementary Figure 3A). We observed that the trajectories of migrating macrophages were longer in the control group compared to the MMG-11 and CU-CPT22 treated groups (Supplementary Figure 3B, D). In addition, the mean speed of macrophage movement in the control, MMG-11 and CU-CPT22 groups was quantified. The mean speed was significantly decreased in the TLR2 inhibitor-treated groups compared with the control group (Supplementary Figure 3C, E). These results show that TLR2 is involved in the process of directed cell migration of macrophages towards mSIOs.

### Transcriptomic profiling reveals inflammatory gene signatures in the infected mSIOs

**Figure 4.**
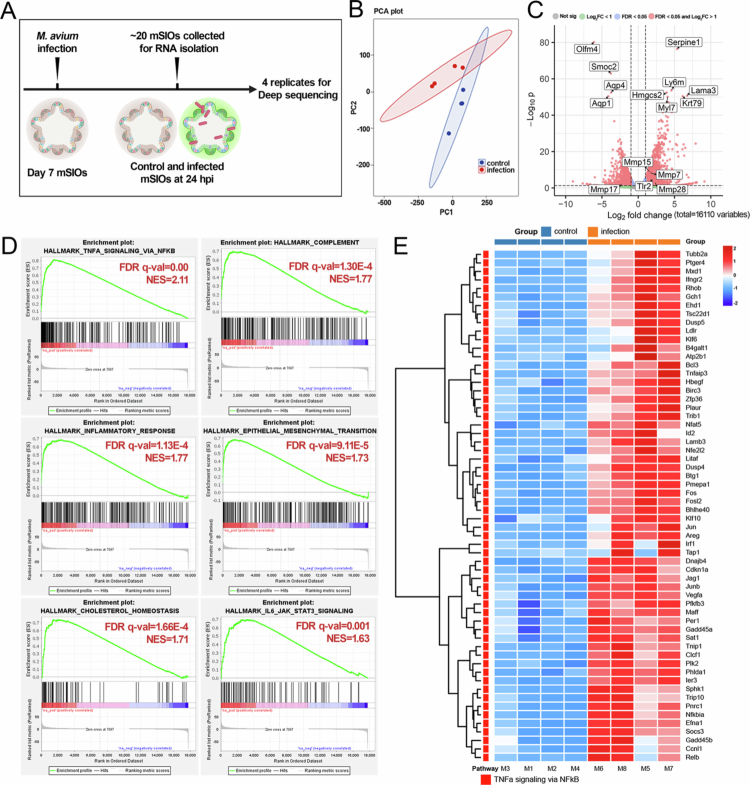
Transcriptomic analysis reveals inflammatory genes induction following *M. avium* infection in mSIOs. (A) Experimental scheme. (B) Principal component analysis. The RNA-seq samples clustered according to their groups, as indicated by the solid ellipses enclosing the samples. (C) Volcano plot showing differentially expressed genes (DEGs) in *M. avium*-infected mSIOs compared to uninfected control mSIOs. (D) Gene Set Enrichment Analysis (GSEA) of hallmark enriched pathways in *M. avium-*infected mSIOs. NES, normalized enrichment scores. (E) Heatmap of gene expression data of the TNFα signaling pathway in the *M. avium*- infected mSIOs. Heatmap showing the expression patterns of selected DEGs. Counts per million (CPM) values were row-normalized (Z-score transformation per gene). Genes and samples were clustered using hierarchical clustering with the Ward.D2 method. Color scale represents row-wise Z-scores, with red indicating higher expression and blue indicating lower expression relative to the gene’s mean.

To identify genes differentially expressed in response to *M. avium* infection in mSIOs, we performed RNA-seq analysis comparing infected and uninfected mSIOs at 24 hpi (Figure 4A). PCA revealed clear separation between the two groups, indicating substantial transcriptional differences induced by infection (Figure 4B). Using a threshold of an FDR-adjusted *p*-value < 0.05 and an absolute log₂ fold change >1, we identified 1,690 upregulated and 1,404 downregulated genes in response to *M. avium* infection (Figure 4C). The top 10 most significantly DEGs (based on FDR *p*-value) are labeled in a volcano plot (Figure 4C, Supplementary Table 1). Notably, *Mmp7*, a matrix metalloproteinase involved in epithelial remodeling and inflammation,^34^ was among upregulated genes. *Tlr2*, a key innate immune receptor that recognizes mycobacterial components,^35^ was also significantly upregulated.

To get an overview of the functional implications of these changes, we performed GSEA using the Hallmark gene set collection. Several immune-related pathways were significantly enriched in the infected mSIOs, including TNF-*α* signaling via NF-κB (top 1-ranked), inflammatory response, and IL-6/JAK/STAT3 signaling (Figure 4D, Supplementary Figure 4). Additionally, pathways such as complement activation, epithelial-mesenchymal transition (EMT), and cholesterol homeostasis were also upregulated, suggesting broader epithelial and metabolic reprogramming (Figure 4D). Figure 4E shows a heatmap of genes associated with TNF-*α* signaling, confirming consistent upregulation across biological replicates of infectious group. We also found increased expression of genes involved in the Toll-like receptor signaling pathway (Supplementary Figure 5), further supporting activation of innate immune responses.

Interestingly, marker genes for intestinal stem cells and transit-amplifying cells were downregulated, whereas genes associated with enterocytes, Paneth cells, goblet cells, and enteroendocrine cells were upregulated (Supplementary Table 2). This shift suggests that *M. avium* infection may suppress stemness while promoting epithelial differentiation, potentially as a defense or repair mechanism. Further GSEA using the KEGG pathway collection revealed significant enrichment of infection-associated pathways, including tight junctions, lysosome, complement and coagulation cascades, retinol metabolism, and arachidonic acid metabolism (Supplementary Figure 6A). Interestingly, many genes within the tight junction pathway were significantly upregulated (Supplementary Figure 6B; Supplementary Table 3), possibly reflecting an epithelial attempt to restore or reinforce barrier integrity in response to bacterial invasion. These transcriptomic changes were accompanied by distinct morphological alterations in infected mSIOs, including increased cellular debris, intercellular spacing, and features of stress such as extended endoplasmic reticulum resembling mitophagy structures^36^ and large multivesicular structures (Supplementary Figure 7).

In summary, *M. avium* infection elicits a robust pro-inflammatory and epithelial remodeling response in mSIOs, characterized by the activation of innate immunity, changes in epithelial cell composition, and upregulation of barrier-related genes. The induction of genes such as *Mmp7* and *Tlr2*, as well as modulation of stem cell and differentiation markers, highlights the relevance of this model in studying CD-like inflammation and epithelial-pathogen interactions.

### Comparative transcriptomic analysis supports overlap with CD-associated inflammation in *M. avium*-infected mSIOs

To determine whether *M. avium* infection in mSIOs has similarities with CD-associated inflammatory responses, we compared our RNA-seq data from *M. avium*-infected mSIOs with a publicly available dataset of germ-free mice colonized with fecal microbiota from either healthy individuals or Crohn’s ileocolitis patients (BioProject PRJNA980747).^20^ This reference dataset includes transcriptomic profiles from the ileum (IL), proximal colon (PC), and distal colon (DC) of the mice humanized with healthy and CD fecal microbiota. Importantly, the CD-humanized mice used for comparison develop mild to moderate colitis *in vivo*, indicating that the analyzed transcriptomes reflect microbiota-induced intestinal inflammation.^20^

**Figure 5.**
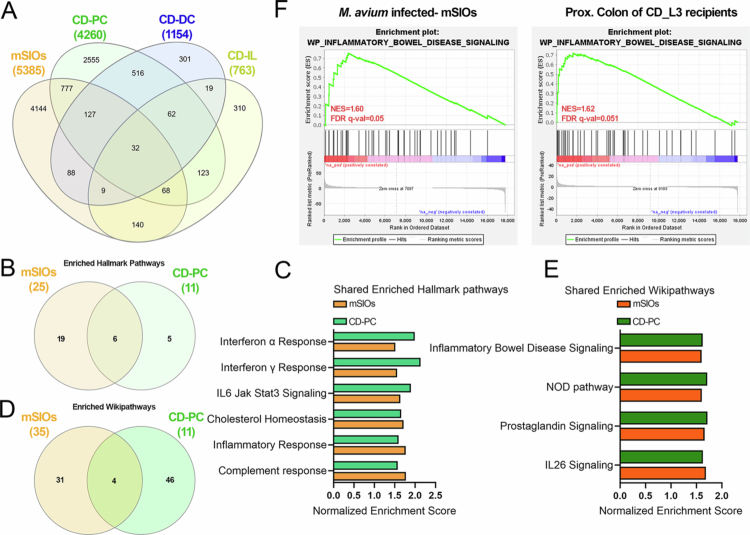
*M. avium***-**infected mSIOs transcriptomes share inflammatory signatures with a Crohn’s Disease- associated mouse model. (A) Venn diagram showing the overlap of DEGs among *M. avium*-infected organoids (refer to mSIOs in this Figure), and the proximal colon (CD-PC), distal colon (CD-DC), and ileum (CD-IL) of humanized mice colonized with fecal microbiota from Crohn’s disease patients (RNA-seq data from BioProject PRJNA980747). DEGs were defined by FDR < 0.05. (B) Venn diagram showing the overlap of significantly enriched Hallmark pathways between the mSIOs and CD-PC groups (FDR < 0.05). (C) Side-by-side bar plot of shared enriched Hallmark pathways in mSIOs and CD-PC groups, based on NES from GSEA. (D) Venn diagram showing the overlap of significantly enriched WikiPathways between mSIOs and CD-PC groups. (E) Side-by-side bar plot of shared enriched WikiPathways in mSIO and CD-PC. (F) GSEA enrichment plots of the “Inflammatory Bowel Disease” pathway in both mSIOs and CD-PC groups, showing coordinated activation of disease-relevant signaling.

A Venn diagram analysis revealed substantial overlap between DEGs from infected mSIOs and those from the IL, PC, and DC tissues of CD microbiota-colonized mice (refer to CD-IL, CD-PC and CD-DC below), indicating shared molecular responses to *M. avium* infected-mSIOs and CD microbiota-induced inflammation (Figure 5A). Among these, the greatest number of shared DEGs was observed between infected mSIOs and the CD-PC, prompting a more detailed comparative analysis between these two groups. Firstly, we performed GSEA using the Hallmark gene set collection to identify commonly activated pathways (Figure 5B, C). Six pathways were significantly enriched in both datasets (Figure 5B). Multiple inflammation-related pathways, including interferon responses, IL6/JAK/STAT3 signaling, and complement response, were significantly enriched in both *M. avium*-infected mSIOs and CD-PC transcriptomes (Figure 5C). To further explore functional similarities, we performed GSEA using the WikiPathways gene set collection (Figure 5D, E). Venn diagram analysis revealed four significantly enriched pathways shared between infected mSIOs and the CD-PC: IL-26 signaling, Prostaglandin signaling, NOD-like receptor signaling, and IBD signaling (Figure 5E). The IBD signaling pathway was significantly upregulated in both datasets, as illustrated by GSEA enrichment plots (Figure 5F, Supplementary Figure 8).

Together, these comparative analyses demonstrate that *M. avium* infection in mSIOs elicits a transcriptional program highly reminiscent of CD-associated inflammation. This supports the utility of this mSIO infection model in studying epithelial-intrinsic responses relevant to human IBD pathogenesis.

### MMP7 is a shared marker of inflammation in *M. avium*-infected mSIOs and CD mouse model

To further study conserved inflammatory markers between *M. avium*–infected mSIOs and the CD mouse model discussed above, we focused on genes commonly upregulated in both datasets. Among the top DEGs in the infected mSIOs, *Mmp7* stood out as one of the most significantly upregulated genes (Figure 4C, Supplementary Table 4).

**Figure 6.**
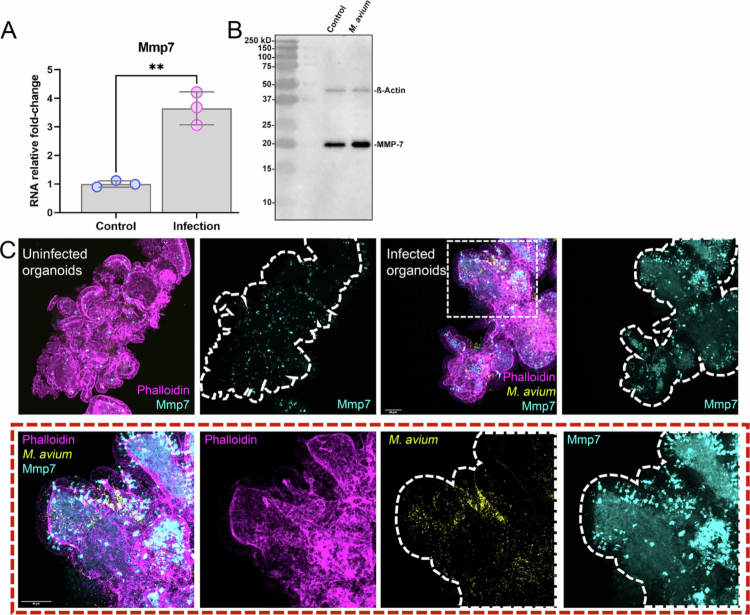
MMP7 is upregulated in *M.* *avium*-infected mSIOs and in the proximal colon of humanized CD mice. (A) qRT-PCR validation of *Mmp7* expression in *M. avium*-infected mSIOs and their uninfected control mSIOs. (B) Western blot shows the higher MMP7 protein level in *M.* *avium-*infected mSIOs compared to the control. (C) MMP7 immunostaining in the *M.* *avium-*infected organoids and the uninfected control. Bottom panel shows higher magnification images (63 × , N.A. 1.4) of *M.* *avium*-infected organoids in the top panel (20 × , N.A. 0.75).

*Mmp7* encodes a matrix metalloproteinase protease involved in epithelial remodeling, mucosal barrier regulation, and innate immune defense, and has previously been implicated in intestinal inflammation and tissue injury.^37^^,^^38^ Analysis of the RNA-seq from humanized mice colonized with CD microbiota showed that *Mmp7* was consistently upregulated in the PC tissue, which has the highest transcriptomic overlap with the gene signature set of infected mSIOs (Figure 5A, Supplementary Table 4). To validate this transcriptome finding, we examined *Mmp7* expression at the mRNA and protein levels in *M. avium*–infected mSIOs. Quantitative RT-PCR confirmed a significant increase in *Mmp7* transcript levels compared to uninfected controls (Figure 6A). This upregulation was further supported by Western blot analysis, which revealed elevated MMP7 protein expression in infected mSIOs (Figure 6B). Immunofluorescence staining demonstrated robust MMP7 localization to the epithelial layer of infected mSIOs, consistent with its known role in epithelial barrier responses (Figure 6C).

Collectively, these findings identify *Mmp7* as a conserved and robust indicator of epithelial inflammation in both *M. avium*–infected mSIOs and CD-like intestinal environments. Its consistent upregulation highlights its potential as a biomarker for infection-associated mucosal inflammation and as a candidate for further mechanistic investigation in the context of IBD.

## Discussion

### Overview of this study

Although the relationship between *M. avium* infections and CD remains unclear, a meta-analysis by Feller et al. reported that *M. avium* is detected more frequently in CD patients than in those with ulcerative colitis, the other major form of IBD.^39^ However, no suitable mammalian whole organism model currently exists to investigate *M. avium*-intestinal infection at early stages. Recently, we have shown that *M. avium* is able to rapidly disseminate into tissues via the gut lumen in a zebrafish larval model (submitted). In this study, we developed an mSIO model to examine the early epithelial responses to *M. avium* infection. Using CLSM and SBF-SEM, we confirmed that *M. avium* can invade and reside within cells of mSIOs. Additionally, in a co-culture system, we observed directed macrophage migration toward infected mSIOs, mimicking early immune cell recruitment. Transcriptomic profiling revealed robust inflammatory gene expression following infection. Comparative analysis with a published CD-humanized mouse model showed significant overlap in gene expression and pathway activation, including shared enrichment of IBD-related signatures. Notably, we identified *MMP7* as a shared inflammation-associated marker in both systems, suggesting its potential role as a molecular link between microbial infection and CD-like intestinal inflammation.

### Establishment of an organoid-based model for *M. avium* intestinal infection

Organoids have become widely used tools for studying infectious diseases due to their ability to recapitulate key features of host tissue architecture and function. However, their application in modeling mycobacterial infections remains limited. Previous studies have used human airway organoids to investigate early-stage infections with *M. abscessus* and *M. tuberculosis.*^40^^,^^41^ Additionally, SIOs derived from bovine or mice tissue have been employed to study MAP infection in the context of JD.^19^^,^^42^^,^^43^ Consistent with these findings, our results demonstrate that *M. avium* can successfully infect cells within mSIOs.^19^ However, the identity of the specific epithelial cell types targeted by MAP remains controversial.^42^^,^^43^ For instance, Baruta et al. reported that MAP preferentially infects M cells in enteroid-derived monolayers.^43^ In contrast, Sangari et al. showed that *M. avium* primarily interacts with enterocytes rather than M cells in an *in vivo* mouse model.^44^ Similarly, Alfituri et al. found that the presence of M cells did not enhance MAP infection in enteroids, and MAP did not preferentially localize to GP2-positive M cells.^42^ In our study, we used an mSIO model without differentiating M cells. Despite the absence of M cells, we observed that *M. avium* was still able to invade the epithelial layer (Figures 1 and 2), suggesting that other epithelial cell types, such as enterocytes, may serve as key entry points for infection. While epithelial cells are not professional phagocytes, they express pattern recognition receptors (PRRs) such as Toll-like receptors (TLRs), enabling them to recognize and internalize microbial pathogens.^45^ Although microbial invasion typically occurs via the apical membrane, increasing evidence suggests that some pathogens can also enter via the basolateral membrane side.^46^^,^^47^ It has been demonstrated that TLR2, TLR4 and TLR5 are preferentially localized on the basolateral surface of small intestinal epithelial cells.^48^^,^^49^ In our study, *Tlr2*—but not *Tlr5*—was significantly upregulated upon infection, consistent with prior evidence that *M. avium* glycopeptidolipids (GPLs) can activate TLR2 signaling.^35^ Taken together, these evidences indicate that TLR2-mediated sensing may contribute to basolateral recognition and internalization of *M. avium*.

### Host cells response to *M. avium* infection

Under homeostatic conditions, intestine-resident macrophages contribute to bacterial clearance while maintaining immune tolerance by avoiding pro-inflammatory cytokine release.^44^^,^^50^ However, during *M. avium* infection, circulating monocytes can be recruited to the intestinal mucosa and differentiate into inflammatory macrophages.^51^ In CD, macrophage numbers are significantly increased in inflamed mucosal areas, contributing to chronic inflammation.^51^ These infiltrating macrophages upregulate iNOS and TNF-*α* expression, leading to compromised epithelial barrier integrity by reducing transepithelial electrical resistance, disrupting tight junction protein expression, and promoting epithelial apoptosis.^52^ Moreover, macrophages are the main cell type of granulomatous lesions in bovine paratuberculosis of the gut.^53^ Although a co-culture system using mSIOs and innate lymphoid cells has been previously described,^33^ co-culture models with macrophages have not yet been reported. Therefore, we established a novel mSIO–macrophage co-culture system to study host–pathogen interactions in early *M. avium* infection (Figure 3A, B). We observed dynamic macrophage clustering and directed migration toward infected mSIOs—consistent with recruitment behavior seen in CD and JD.^51^^-^^53^ When macrophages were pretreated with TLR2 inhibitors before co-culture, both displacement and mean migration speed were significantly reduced (Supplementary Figure 3), supporting a role for TLR2-dependent signaling in cellular responses to *M.* *avium-*infected mSIOs. It is consistent to our previous work in zebrafish. We have shown that *tlr2* regulates leukocyte behavior during wounding and mycobacterial infection.^54^^,^^55^ It should be noted that this co-culture system is intentionally reductionist and focuses on epithelial–macrophage interactions. Other key immune populations implicated in Crohn’s disease, such as dendritic cells, T cell subsets (Th1, Th17, Treg), and ILC3 are not included. These additional arms of immunity will need to be incorporated in future iterations of the model to more fully capture the complexity of CD-associated immune dysfunction.

Unexpectedly, we also observed that epithelial cells of mSIOs were able to sense and move to the presence of *M. avium* (Figure 1D). *M. avium*–induced basolateral movement of epithelial cells is notable and may involve several sensing mechanisms. Enterocytes, like M cells, express integrins on their basolateral membrane.^56^^,^^57^ *M. avium* can engage integrins on macrophages during entry.^58^ However, there is no evidence that enterocyte integrins directly bind *M. avium*, and integrins are not chemotactic receptors. Thus, they likely contribute only to adhesion or cytoskeletal reorganization rather than directional migration. It has been demonstrated that *M. avium* itself secretes an MCP-1–like chemotactic factor to recruit THP-1 cells, monocytes and macrophages.^59^ However, the corresponding receptor CCR2 was not detected in our RNA-seq dataset, making this pathway unlikely in *M.* *avium-*infected mSIOs. Based on current evidence, the most plausible explanation is that the observed movement represents reorganization of the epithelial cytoskeleton driven by TLR2-dependent sensing, possibly acting together with integrin-mediated adhesion dynamics. Alternatively, *M. avium* may release yet-unidentified chemoattractant capable of activating chemokine receptors on the basolateral side of epithelial cells. Future mechanistic studies will be required to dissect these pathways in detail.

Our study characterizes early epithelial responses to *M. avium* infection; however, the intracellular fate of the bacteria and the downstream host cell-death pathways remain incompletely defined. Although autophagy- and cell-death–related pathways were not significantly enriched by GSEA, serial block-face SEM revealed morphological signs of epithelial stress, including cellular debris and features of stress such as extended endoplasmic reticulum resembling mitophagy (Supplementary Figure 7). To explore this further, we examined significantly regulated genes (FDR *p*-value < 0.05) within apoptosis, ferroptosis, and mitophagy pathways (Supplementary Tables 5–7). Notably, *Dram2*, a DNA-damage–regulated autophagy modulator implicated in host defense against *M. tuberculosis*, was significantly upregulated in infected mSIOs (Supplementary Table 7). Although few canonical pyroptosis-associated genes were differentially expressed, the pyroptosis executioner *Gsdmd* was significantly induced in *M. avium*–infected mSIOs. Furthermore, the upstream NOD signaling pathway—an established regulator of inflammatory cell death and autophagy—was significantly enriched in infected mSIOs (Figure 5E). Together, these transcriptional and ultrastructural findings indicate that *M. avium* infection activates epithelial stress-response pathways, including autophagy-related processes and pyroptosis-associated signaling, highlighting potential mechanisms that merit further mechanistic investigation.

### Parallels between *M. avium* infection and CD inflammation

Both JD and CD are characterized by chronic intestinal inflammation. Among the key mediators of this inflammatory environment is TNF-*α*, which plays a central role in driving the mucosal inflammation and tissue damage observed in CD patients.^60^ In addition, the IL-6/JAK/STAT3 signaling pathway, while essential for maintaining intestinal epithelial integrity and regeneration, has been implicated in perpetuating inflammation and contributing to disease severity in IBD.^61^ In our study, GSEA of *M. avium*-infected mSIOs revealed that inflammatory pathways, including TNF-*α* signaling via NF-κB and IL-6/JAK/STAT3 signaling, were significantly upregulated (Figure 4D, E). Consistently, we also observed significant enrichment of IL-6/JAK/STAT3 signaling in the proximal colon of humanized mice colonized with CD microbiota (CD-PC) (Figure 5C). Our result suggests that *M. avium* infection can trigger potent inflammatory cascades even in the absence of immune cells, highlighting the epithelial layer as an early responder in disease pathogenesis.

We also found upregulation of EMT pathways in infected mSIOs (Figure 4D). EMT is a process by which epithelial cells lose polarity and intercellular adhesion, acquiring mesenchymal features that increase migratory capacity and resistance to apoptosis.^62^ Although EMT is important in development and wound healing, its persistent activation has been associated with fibrosis, cancer progression, and disruption of epithelial barriers.^62^ Notably, IL-6/STAT3 signaling has been shown to induce EMT in colorectal cancer,^63^ raising the possibility that chronic *M. avium* infection could initiate similar processes in the intestinal epithelium. Interestingly, we also observed upregulation of goblet cell marker genes in infected mSIOs (Supplementary Table 2). Prior work has shown that MAP can invade goblet cells and cause goblet cell hyperplasia, a lesion associated with both JD and early-stage colorectal carcinogenesis.^64^ These observations raise the intriguing hypothesis that *M. avium* infection may contribute not only to chronic inflammation but also to epithelial remodeling processes associated with neoplastic transformation.

Additionally, we found that many genes from tight junction pathway were also upregulated (Supplementary Figure 6B, Supplementary Table 3). While this may initially appear contradictory, it could reflect a compensatory response by epithelial cells to preserve barrier function under stress at *M. avium* early infection stage. Notably, we did not detect overt barrier disruption in our system, suggesting that these molecular changes precede measurable functional damage. Taken together, our findings suggest that *M. avium* triggers a transcriptional program in intestinal epithelial cells characterized by inflammation, altered differentiation, and potential precancerous remodeling. Future studies should explore the long-term consequences of *M. avium* infection *in vivo*, including the persistence of EMT features, barrier integrity, and links to colorectal cancer risk.

*MMP7* is notably elevated in inflamed intestinal tissue of IBD patients.^65^^,^^66^ Crucially, functional studies have shown that MMP7 degrades Claudin-7, a key tight junction protein, leading to increased epithelial permeability and barrier dysfunction.^38^ Furthermore, elevated MMP7 expression is specifically associated with epithelial cells lining ulcerated mucosa in both CD and ulcerative colitis, indicating its involvement in tissue remodeling and damage at the site of injury.^34^^,^^67^ In our study, the transcriptional response of *M.* *avium-*infected mSIOs showed the strongest overlap with the PC transcriptome of mice colonized with CD- associated microbiota. Although this is remarkable given that our mSIOs are derived from the ileum, this similarity may reflect general inflammation-driven transcriptional programs rather than a strict Paneth-cell-like signature. Nevertheless, the Paneth-like differentiation hypothesis proposed by Sheikh et al.^20^ remains an interesting possibility. This is in accordance with the observation that MMP7 is strongly increased in the PC samples, whereas it is normally a Paneth cell-restricted matrix metallopeptidase associated with colonic inflammation in both CD and UC as well as colorectal cancer.^38^^,^^66^ Thus, MMP7 likely serves as a pivotal mediator linking microbial challenge to epithelial barrier compromise and inflammation. Given its consistent upregulation across models and established pathophysiological roles, MMP7 merits further investigation. Future work should focus on testing MMP7 inhibition—using genetic knockouts or selective inhibitors—in mSIO systems and *in vivo* models of *M. avium* infection. Such studies could determine whether targeting MMP7 can preserve epithelial function and mitigate infection-driven mucosal damage.

Paneth cells are specialized epithelial cells located at the base of intestinal crypts, particularly in the small intestine.^68^^,^^69^ They play a critical role in maintaining mucosal homeostasis by secreting antimicrobial peptides such as *α*-defensins (cryptdins in mice), lysozyme, and other defense molecules, which help regulate the gut microbiota and reinforce the intestinal barrier.^68^ Paneth cell dysfunction has been strongly associated with the pathogenesis of CD, especially in ileal CD.^68^^,^^70^ Mutations in genes like NOD2—a well-established CD susceptibility gene, which was also upregulated in our study—can impair Paneth cell function and autophagy, leading to reduced secretion of antimicrobial factors, abnormal granule morphology, and increased vulnerability to dysbiosis and microbial invasion.^71^ Interestingly, we observed upregulation of Paneth cell-associated genes, including *Lyz1* and *Mmp7*, in *M. avium*-infected mSIOs (Supplementary Table 2). This may represent a compensatory epithelial response aimed at enhancing antimicrobial defenses. Given the established role of Paneth cell dysfunction in CD pathogenesis, future studies should assess whether *M. avium* infection alters Paneth cell granule antimicrobial peptide processing, or stem cell niche signaling, thereby contributing to sustained intestinal inflammation.

### Limitations and future perspectives

Although this study provides a foundational model for examining early epithelial and macrophage responses to *M. avium* infection, it has several limitations. First, our organoid system captures only the acute phase of infection and therefore does not model the chronic, relapsing inflammation characteristic of Crohn’s disease. Second, the co-culture includes only macrophages, without other key immune populations such as dendritic cells, T cells, ILC3, or neutrophils, limiting our ability to explore broader immune network interactions. Third, the organoids were derived from wild-type mice and do not incorporate CD-relevant genetic susceptibility variants (e.g., *Nod2*, *Atg16l1*), which may shape host responses to *M. avium*. Finally, we modeled infection with a single bacterial species, while CD arises in a complex polymicrobial environment and dysbiotic microbiota.

Despite these limitations, the model provides a powerful platform for dissecting early host–pathogen interactions under controlled conditions. Future work can extend this system by introducing chronic or repeated infection, incorporating genetically modified organoids carrying CD-associated variants, and expanding the co-culture to include additional immune cell types. Human intestinal organoids co-cultured with human macrophages would further enhance translational relevance. Mechanistic studies on autophagy, pyroptosis, and bacterial persistence—guided by the transcriptional signatures identified here—may also reveal how *M. avium* interacts with epithelial defense pathways. Together, these extensions will help define whether *M. avium* acts as a trigger, amplifier, or bystander in CD-like inflammation.

## Supplementary Material

Supplementary_Materials_Revised_Final clean.docx Supplementary_Materials_Revised_Final clean.docx

## Data Availability

The RNA-seq dataset generated and analyzed during this study is available at the NCBI Gene Expression Omnibus (GEO) under the accession number: GSE305886. The repository of the images in this study is available at Figshare: 10.6084/m9.figshare.29996368.
