# Supplementary material for: Responses of intestinal organoids to infection by Mycobacterium avium resemble symptoms observed in Crohn’s disease
Source: Gut Microbes. 2026 Feb 13;18(1):2630483. doi: 10.1080/19490976.2026.2630483 (PMC12915785; doi:10.1080/19490976.2026.2630483)
Supplement: Supplementary_Materials_Revised_Final clean.docx [file KGMI_A_2630483_SM6306.docx]

**Supplementary Materials**

**
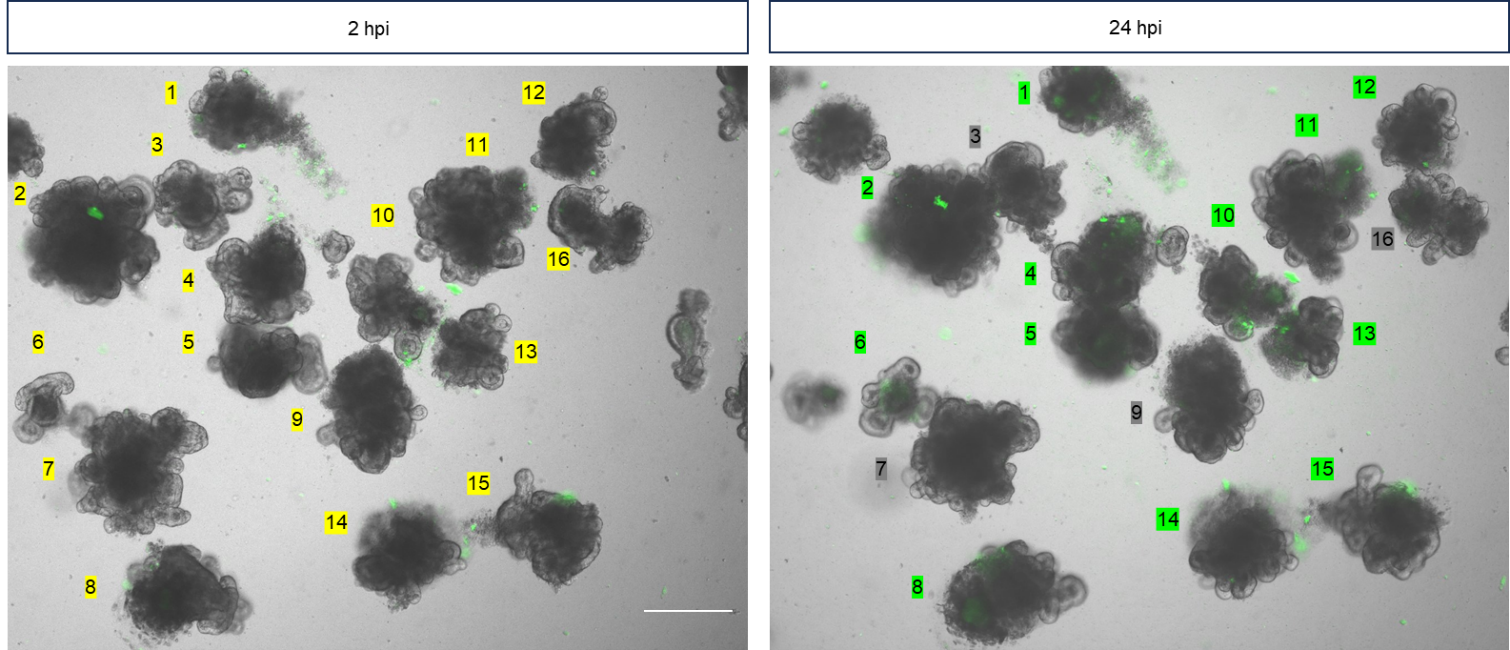
**

**Supplementary Figure 1.** Overview of *M. avium* infection dynamics in mSIOs between 2 and 24 hpi.


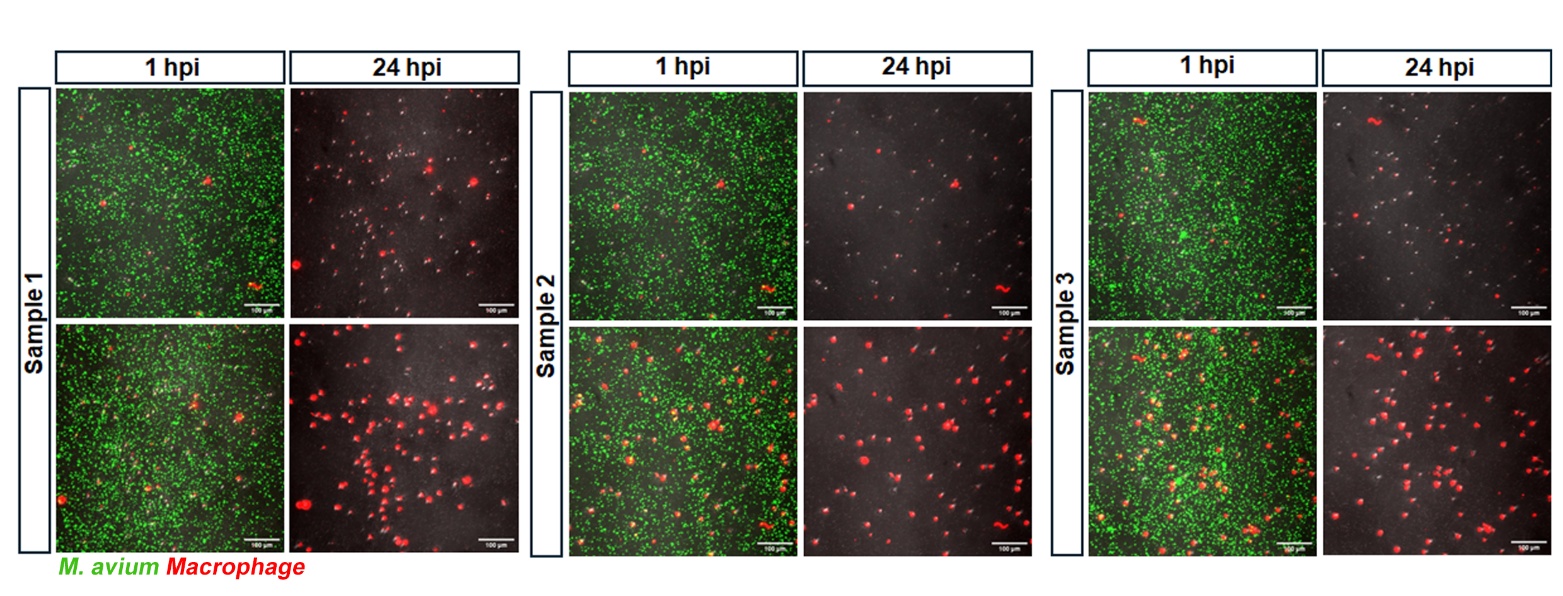


**Supplementary Figure 2.** Confocal images of *M. avium-* infected RAW267.4 macrophages. Macrophages were culture in BME with DMEM medium. Scale bar: 100µm.


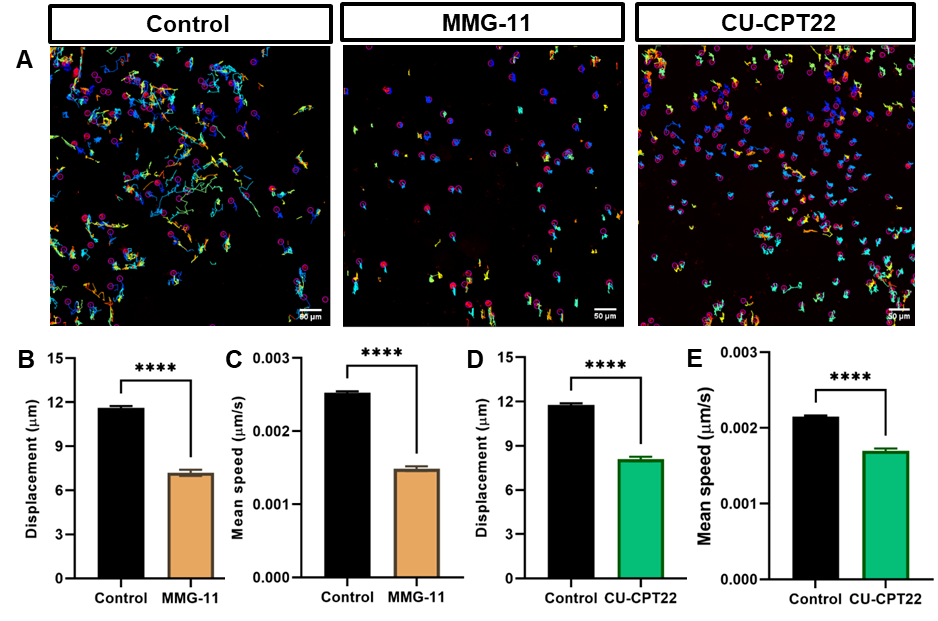


**Supplementary Figure 3.** TLR2 regulates macrophages getting cluster around the *M. avium*-infected mSIOs. (A) Representative images of the macrophage tracks in *M. avium*-infected mSIOs and macrophage co-culture system. Red circle represents individual macrophage. Scale bar: 50 µm. (B, C) Quantification of macrophage migration displacement and migration mean speed in control and MMG-11 treatment group. (D, E) Quantification of macrophage migration displacement and migration mean speed in control and CU-CPT22 treatment group. Statistical significance of differences was determined by unpaired t-test for comparison between Control and TLR2 inhibitor treatment groups, ****, P < 0.0001.

**
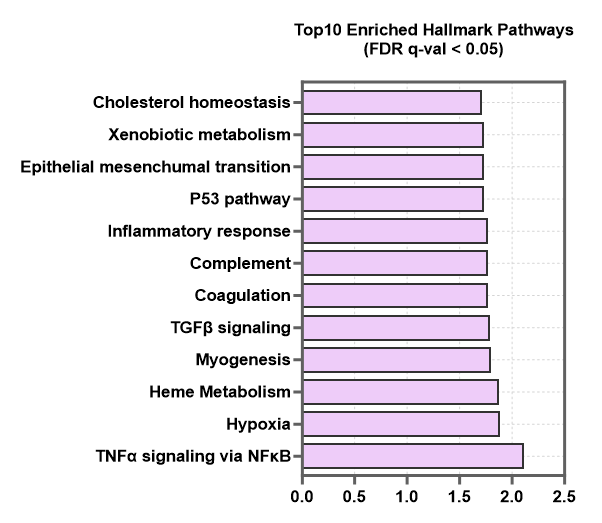
**

**Supplementary Figure 4.** Top 10 enriched Hallmark Pathways in *M. avium*-infected mSIOs.

**
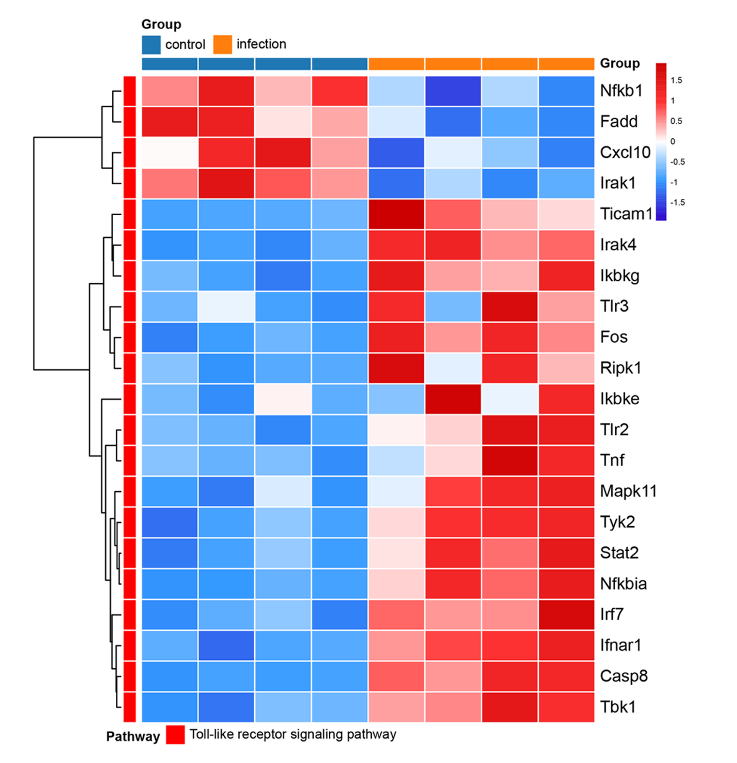
**

**Supplementary Figure 5.** Heatmap of gene expression data of the TLR2 signaling pathway in the *M. avium*- infected mSIOs.


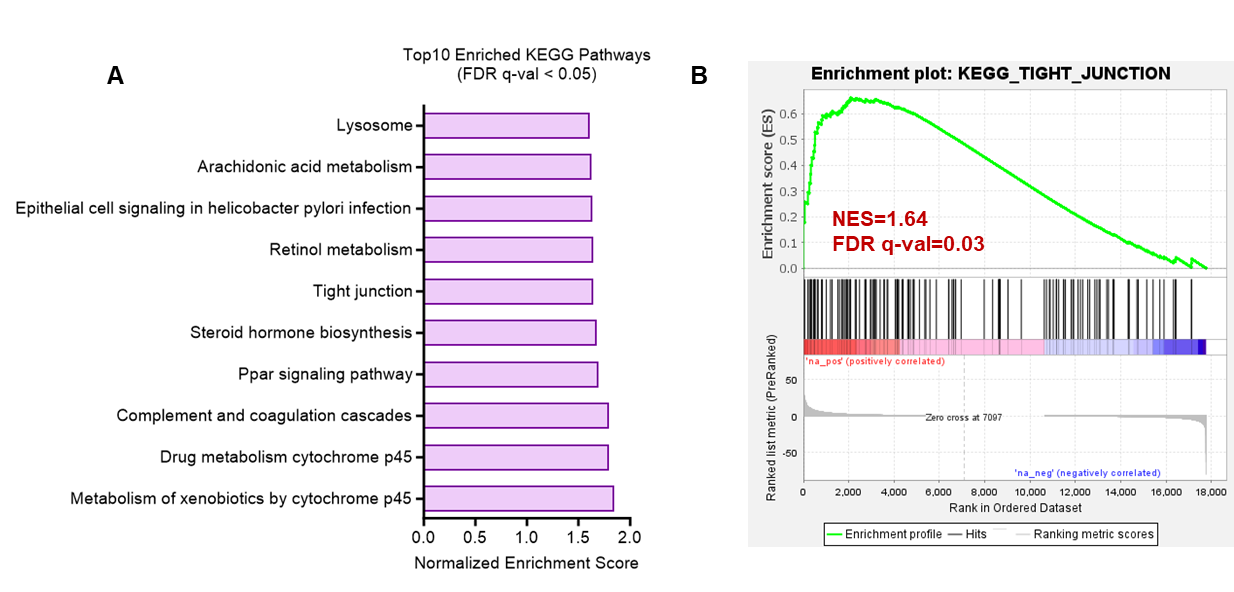


**Supplementary Figure 6.** DEGs of *M. avium* infected- mSIOs are significantly enriched in tight junction pathway by GSEA analysis. (A) Top 10 enriched KEGG pathways in *M. avium*-infected mSIOs. (B) Enrichment plot of tight junction. Please see the supplementary table 3 for details of DEGs in tight junction pathway.


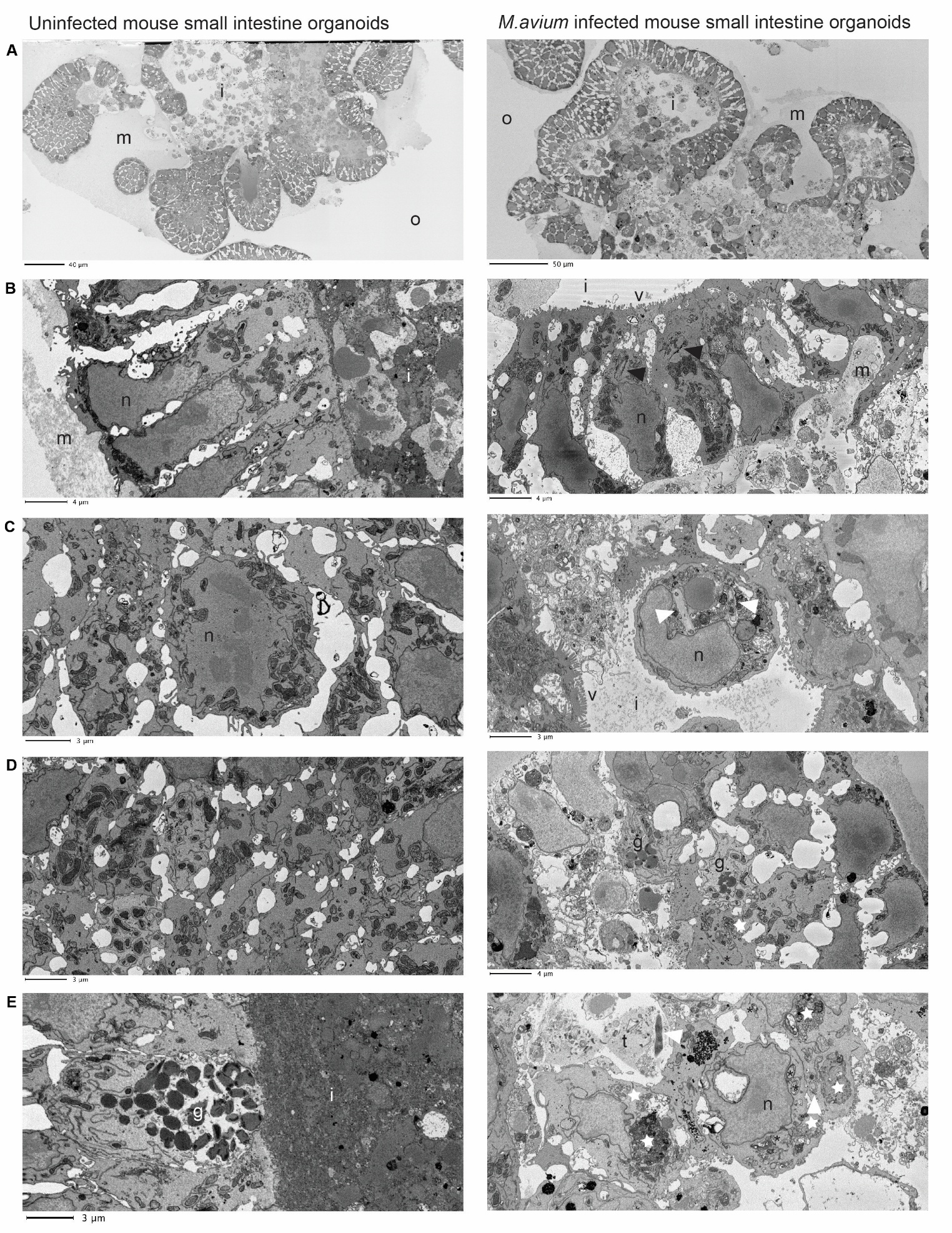


**Supplementary Figure 7.** Comparisons of the morphology between the control and *M. avium*-infected mSIOs via SFB-SEM. Selected solid block face SEM snapshots of uninfected (left row) and *M. avium* infected (tight row) mSIO.

In (A) column, it shows overview of organoids with the outside (o) and inside (i) of the organoids, as well as the Matrigel (m, light grey) annotated. The organoids (main dark features) in the infected mSIOs appear to have wider spacings between the cells that in uninfected mSIOs and there appear to be more loose and apoptotic cells inside the organoids.

In (B) column, it shows a few cells of the epithelial layer with the basolateral side and the nucleus positioned towards the Matrigel (m) and the mitochondria and microvilli (v) position towards the apical membrane (i). The lumen of the uninfected cells are typically filled with dark (proteinaceous) material or loose cells, while in *M. avium* infected organoids the lumen is typically filled with cellular debris and apoptotic cells.

In (C) column, while we observed several dividing cells and no infected cells in the control organoids, in the infected organoids no dividing cells were observed and several cells containing bacteria (white arrowheads) were found, though most cells were not infected.

In (D) column, uninfected cells showed a uniform morphology with little variation between the cells. Mitochondria were typically surrounded by endoplasmic reticulum, which likely plays a role in mitochondrial regulation, signaling and remodeling (left panel). The morphological variation in infected mSIOs was much larger (right panel).

In (E) column, the endoplasmic reticulum was dilated, highly elongated, dark spots were present an it formed extensive sheets and long (multilamellar) tubes (asterisk). Extensive multivesicular vesicles/vacuoles were formed (white stars), a well as dark granular patches, located on the surface of vesicular structures, which highly resemble mitophagy. Also, increased detachment between cells, disintegrating cells and cellular debris were observed. These morphological changes were omnipresent and not only in (the few) infected cells (white arrowheads). Occasional Paneth cells with characteristic granules (g) were observed in both infected and uninfected cells (bottom panels). These granules either have a uniformly dark appearance or a light grey appearance containing a darker torus, aligning the outside. Torus-shapes structures (t) are occasionally present (without being surrounded by a full granule) outside the cells in uninfected mSIOs, but omni present in infected mSIOs also near bacteria (lower right panel), suggesting that the presence of *M. avium* activates secretion of Paneth cell granules.


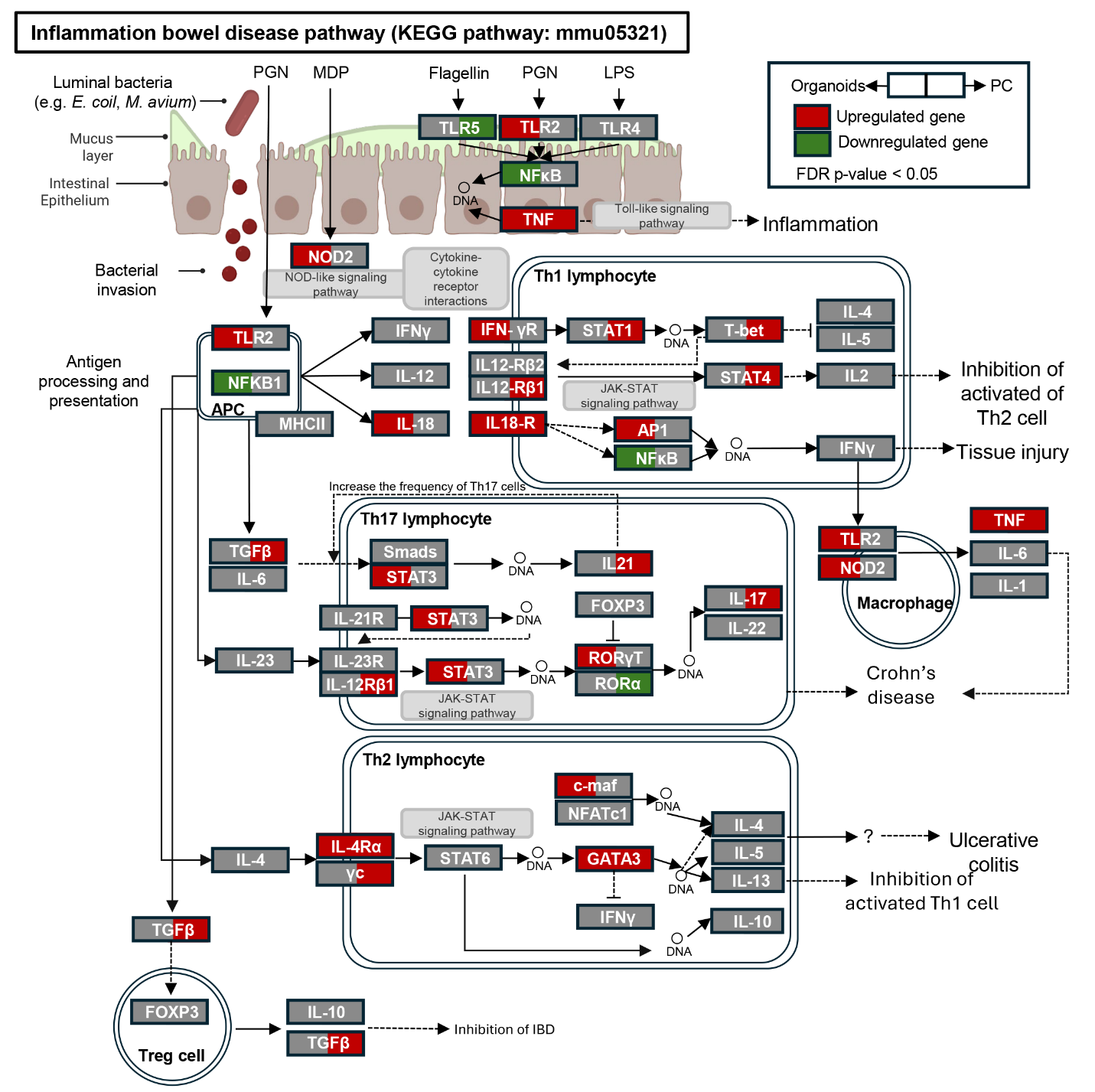


**Supplementary Figure 8.** The DEGs expression profile of *M.avium* infected organoids and PC of humanized mice colonized with fetal microbiota from Crohn’s disease patient in the Inflammatory Bowel Disesae pathway. The pathway is adapted with KEGG pathway (mmu05321). Upregulated genes are highlighted in red, and downregulated genes are highlighted in green.

**Supplementary Table 1. Top 10 differentially expressed genes (DEGs) in *M. avium* infected mSIOs.**

| **Gene** | **Description** | **ENSEMBL ID** | | **FDR p-value** | **Log2 Fold Change** |
| --- | --- | --- | --- | --- | --- |
| Olfm4 | olfactomedin 4 | | ENSMUSG00000022026 | 6.04E-81 | -6.12 |
| Serpine1 | serine (or cysteine) peptidase inhibitor,  clade E, member 1 | | ENSMUSG00000037411 | 4.00E-77 | 5.36 |
| Smoc2 | SPARC related modular calcium binding 2 | | ENSMUSG00000023886 | 1.07E-63 | -3.78 |
| Ly6m | lymphocyte antigen 6 family member M | | ENSMUSG00000063522 | 2.09E-54 | 4.57 |
| Hmgcs2 | 3-hydroxy-3-methylglutaryl-Coenzyme A synthase 2 | | ENSMUSG00000027875 | 3.46E-53 | 3.92 |
| Aqp4 | aquaporin 4 | | ENSMUSG00000024411 | 3.46E-53 | -3.74 |
| Lama3 | laminin, alpha 3 | | ENSMUSG00000024421 | 1.88E-51 | 6.73 |
| Krt79 | keratin 79 | | ENSMUSG00000061397 | 3.69E-51 | 6.13 |
| Aqp1 | aquaporin 1 | | ENSMUSG00000004655 | 3.77E-51 | -4.12 |
| Myl7 | myosin, light polypeptide 7, regulatory | | ENSMUSG00000020469 | 8.02E-51 | 4.05 |

**Supplementary Table 2. Expression of marker genes on cells of small intestine**

| **Cell type** | **Marker gene** | **Description** | **ENSEMBL ID** | **FDR p-value** | **Log2 Fold Change** |  |
| --- | --- | --- | --- | --- | --- | --- |
| LGR5^+^ CBC cell | Lgr5 | leucine rich repeat containing G protein coupled receptor 5 | ENSMUSG00000020140 | 7.7E-18 | -2.40 | 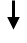 |
|  | Ascl2 | achaete-scute family bHLH transcription factor 2 | ENSMUSG00000009248 | 0.10175 | -0.54 | 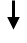 |
|  | Olfm4 | olfactomedin 4 | ENSMUSG00000022026 | 6.04E-81 | -6.12 | 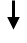 |
|  | Sox9 | SRY (sex determining region Y)-box 9 | ENSMUSG00000000567 | 0.00025 | -0.98 | 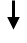 |
| Transit- amplifying cell | Mki67 | antigen identified by monoclonal antibody Ki 67 | ENSMUSG00000031004 | 1.80E-06 | -1.30 | 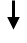 |
|  | Pcna | proliferating cell nuclear antigen | ENSMUSG00000027342 | 5.36E-05 | -1.05 | 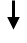 |
| Enterocyte | Vil1 | villin 1 | ENSMUSG00000026175 | 0.00199 | 0.88 | 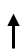 |
| Paneth cell | Lyz1 | lysozyme 1 | ENSMUSG00000069515 | 0.00081 | 0.91 | 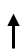 |
|  | Mmp7 | matrix metallopeptidase 7 | ENSMUSG00000018623 | 4.70E-09 | 1.65 | 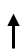 |
| Goblet cell | Muc2 | mucin 2 | ENSMUSG00000025515 | 0.04347 | 0.98 | 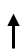 |
| Enteroendocrine cell | ChgA | chromogranin A | ENSMUSG00000021194 | 1.17E-18 | 2.51 | 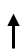 |
| Tuft cell | Dclk1 | doublecortin-like kinase 1 | ENSMUSG00000027797 | 0.91383 | 0.09 | 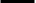 |

Note: LGR5^+^ CBC cell: LGR5+ crypt base columnar cells; Quiescent ISC: Quiescent intestinal stem cells; M cell: microfold cell.

**Supplementary Table 3.** Expression of genes in tight junction pathway

| **Gene** | **Description** | **ENSEMBL ID** | **FDR p-value** | **Log2 Fold Change** |
| --- | --- | --- | --- | --- |
| Myl7 | myosin, light polypeptide 7, regulatory | ENSMUSG00000020469 | 8.01E-51 | 4.05 |
| Cldn4 | claudin 4 | ENSMUSG00000047501 | 2.47E-23 | 3.00 |
| Cldn23 | claudin 23 | ENSMUSG00000055976 | 2.77E-13 | 2.40 |
| Amotl1 | angiomotin-like 1 | ENSMUSG00000013076 | 6.53E-12 | 3.14 |
| Myh14 | myosin, heavy polypeptide 14 | ENSMUSG00000030739 | 4.81E-11 | 1.70 |
| Crb3 | crumbs family member 3 | ENSMUSG00000044279 | 1.15E-10 | 1.39 |
| Epb41l1 | erythrocyte membrane protein band 4.1 like 1 | ENSMUSG00000027624 | 9.81E-10 | 1.48 |
| F11r | F11 receptor | ENSMUSG00000038235 | 1.31E-08 | 1.48 |
| Prkcd | protein kinase C, delta | ENSMUSG00000021948 | 4.01E-08 | 1.32 |
| Actb | actin, beta | ENSMUSG00000029580 | 3.84E-07 | 1.30 |
| Cgn | cingulin | ENSMUSG00000068876 | 7.31E-07 | 1.22 |
| Cttn | cortactin | ENSMUSG00000031078 | 4.96E-06 | 1.07 |
| Igsf5 | immunoglobulin superfamily, member 5 | ENSMUSG00000000159 | 7.99E-06 | 1.08 |
| Csnk2a2 | casein kinase 2, alpha prime polypeptide | ENSMUSG00000046707 | 5.29E-05 | 1.13 |
| Llgl2 | LLGL2 scribble cell polarity complex component | ENSMUSG00000020782 | 0.00022 | 0.85 |
| Cldn3 | claudin 3 | ENSMUSG00000070473 | 0.00036 | 1.03 |
| Actn4 | actinin alpha 4 | ENSMUSG00000054808 | 0.00149 | 0.86 |
| Ppp2r2d | rotein phosphatase 2, regulatory subunit B, delta | ENSMUSG00000041769 | 0.00211 | 0.71 |
| Rab3b | RAB3B, member RAS oncogene family | ENSMUSG00000003411 | 0.00299 | 2.43 |
| Pals1 | protein associated with LIN7 1, MAGUK family member | ENSMUSG00000021112 | 0.00338 | 0.75 |
| Myl12b | myosin, light chain 12B, regulatory | ENSMUSG00000034868 | 0.00428 | 0.74 |
| Ctnna1 | catenin alpha 1 | ENSMUSG00000037815 | 0.00571 | 0.76 |
| Pard6b | par-6 family cell polarity regulator beta | ENSMUSG00000044641 | 0.00698 | 0.75 |
| Tjp3 | tight junction protein 3 | ENSMUSG00000034917 | 0.00727 | 0.69 |
| Tjp2 | tight junction protein 2 | ENSMUSG00000024812 | 0.00834 | 0.67 |
| Gnai2 | G protein subunit alpha i2 | ENSMUSG00000032562 | 0.01030 | 0.69 |
| Afdn | afadin, adherens junction formation factor | ENSMUSG00000068036 | 0.01186 | 0.65 |
| Magi1 | membrane associated guanylate kinase, WW and PDZ domain containing 1 | ENSMUSG00000045095 | 0.01228 | 0.65 |
| Cldn7 | claudin 7 | ENSMUSG00000018569 | 0.02042 | 0.68 |
| Prkca | protein kinase C, alpha | ENSMUSG00000050965 | 0.03168 | 0.56 |
| Jam2 | junction adhesion molecule 2 | ENSMUSG00000053062 | 0.05051 | 2.37 |
| Rras2 | related RAS viral (r-ras) oncogene 2 | ENSMUSG00000055723 | 0.05451 | 0.49 |

**Supplementary Table 4.** Expression of Mmp7 in M. avium infected- mSIOs and different tissues of humanized mice colonized with fetal microbiota from Crohn’s disease patients.

| **Samples** | **Log₂ fold change** | **Fold change** | **adj p-value** |
| --- | --- | --- | --- |
| mSIOs | 1.64 | 3.13 | 4.70E-09 |
| CD-DC | 5.55 | 46.89 | 1.8E-06 |
| CD-PC | 1.51 | 2.85 | 2.6E-08 |
| CD-IL | 0.35 | 1.27 | 0.56 |

**Note:** CD-DC, the distal colon of humanized mice colonized with fetal microbiota from Crohn’s disease patients. CD-PC, the proximal colon of humanized mice colonized with fetal microbiota from Crohn’s disease patients. CD-IL, the ileum of humanized mice colonized with fetal microbiota from Crohn’s disease patients

**Supplementary Table 5.** Expression of genes in apoptosis pathway

| **Gene** | **Description** | **ENSEMBL ID** | **FDR p-value** | **Log2 Fold Change** |
| --- | --- | --- | --- | --- |
| Capn2 | calpain 2 | ENSMUSG00000026509 | 1.9E-19 | 2.34 |
| Fos | FBJ osteosarcoma oncogene | ENSMUSG00000021250 | 1.1E-17 | 2.19 |
| Parp1 | poly (ADP-ribose) polymerase family, member 1 | ENSMUSG00000026496 | 2.93E-16 | -1.87 |
| Tnfsf10 | tumor necrosis factor (ligand) superfamily, member 10 | ENSMUSG00000039304 | 1.63E-15 | -3.07 |
| Casp2 | caspase 2 | ENSMUSG00000029863 | 4.38E-15 | 1.81 |
| Nfkbia | nuclear factor of kappa light polypeptide gene enhancer in B cells inhibitor, alpha | ENSMUSG00000021025 | 5.49E-14 | 1.88 |
| Lmnb1 | lamin B1 | ENSMUSG00000024590 | 9.72E-12 | -1.60 |
| Bcl2 | B cell leukemia/lymphoma 2 | ENSMUSG00000057329 | 1.03E-11 | -2.06 |
| Pmaip1 | phorbol-12-myristate-13-acetate-induced protein 1 | ENSMUSG00000024521 | 1.00E-11 | 1.56 |
| Tuba1b | tubulin, alpha 1B | ENSMUSG00000023004 | 2.23E-10 | -1.66 |
| Birc5 | baculoviral IAP repeat-containing 5 | ENSMUSG00000017716 | 3.71E-09 | -1.46 |
| Ctsd | cathepsin D | ENSMUSG00000007891 | 1.39E-08 | 1.70 |
| Jun | jun proto-oncogene | ENSMUSG00000052684 | 2.27E-08 | 1.44 |
| Birc3 | baculoviral IAP repeat-containing 3 | ENSMUSG00000032000 | 4.97E-08 | 1.46 |
| Casp8 | caspase 8 | ENSMUSG00000026029 | 7.19E-08 | 1.24 |
| Actb | actin, beta | ENSMUSG00000029580 | 3.84E-07 | 1.30 |
| Bcl2l1 | BCL2-like 1 | ENSMUSG00000007659 | 1.09E-06 | 1.09 |
| Lmna | lamin A | ENSMUSG00000028063 | 3.10E-06 | 1.15 |
| Trp53 | transformation related protein 53 | ENSMUSG00000059552 | 4.15E-06 | -1.08 |
| Ikbkg | inhibitor of kappaB kinase gamma | ENSMUSG00000004221 | 4.69E-06 | 1.02 |
| Ctsh | cathepsin H | ENSMUSG00000032359 | 7.37E-06 | 1.06 |
| Mapk3 | mitogen-activated protein kinase 3 | ENSMUSG00000063065 | 2.06E-05 | 1.05 |
| Ctsl | cathepsin L | ENSMUSG00000021477 | 2.49E-05 | 1.34 |
| Parp3 | poly (ADP-ribose) polymerase family, member 3 | ENSMUSG00000023249 | 3.35E-05 | 1.24 |
| Htra2 | HtrA serine peptidase 2 | ENSMUSG00000068329 | 3.40E-05 | -0.97 |
| Fas | Fas cell surface death receptor | ENSMUSG00000024778 | 5.31E-05 | 1.95 |
| Atm | ataxia telangiectasia mutated | ENSMUSG00000034218 | 5.72E-05 | -1.55 |
| Map2k2 | mitogen-activated protein kinase kinase 2 [S | ENSMUSG00000035027 | 2.31E-04 | 0.87 |
| Gadd45a | growth arrest and DNA-damage-inducible 45 alpha | ENSMUSG00000036390 | 2.66E-04 | 0.92 |
| Bcl2l11 | BCL2 like 11 | ENSMUSG00000027381 | 4.16E-04 | 0.91 |
| Tradd | TNFRSF1A-associated via death domain | ENSMUSG00000031887 | 4.97E-04 | 0.84 |

| **Gene** | **Description** | **ENSEMBL ID** | **FDR p-value** | **Log2 Fold Change** |
| --- | --- | --- | --- | --- |
| Gadd45b | growth arrest and DNA-damage-inducible 45 beta | ENSMUSG00000015312 | 1.68E-03 | 0.79 |
| Dffb | DNA fragmentation factor, beta subunit | ENSMUSG00000029027 | 3.47E-03 | 0.84 |
| Cycs | cytochrome c, somatic | ENSMUSG00000063694 | 3.50E-03 | 0.70 |
| Fadd | Fas associated via death domain | ENSMUSG00000031077 | 4.10E-03 | -0.85 |
| Tubal3 | tubulin, alpha-like 3 | ENSMUSG00000021216 | 5.58E-03 | 0.76 |
| Tnfrsf1a | tumor necrosis factor receptor superfamily, member 1a | ENSMUSG00000030341 | 5.75E-03 | 0.69 |
| Birc2 | baculoviral IAP repeat-containing 2 | ENSMUSG00000057367 | 7.06E-03 | 0.72 |
| Bak1 | BCL2-antagonist/killer 1 | ENSMUSG00000057789 | 7.78E-03 | 0.65 |
| Pik3r1 | phosphoinositide-3-kinase regulatory subunit 1 | ENSMUSG00000041417 | 8.38E-03 | -0.70 |
| Bax | BCL2-associated X protein | ENSMUSG00000003873 | 8.93E-03 | 0.77 |
| Nfkb1 | nuclear factor of kappa light polypeptide gene enhancer in B cells 1, p105 | ENSMUSG00000028163 | 9.53E-03 | -0.65 |
| Mapk8 | mitogen-activated protein kinase 8 | ENSMUSG00000021936 | 9.93E-03 | 0.63 |
| Aifm1 | apoptosis-inducing factor, mitochondrion-associated 1 | ENSMUSG00000036932 | 0.01 | -0.62 |
| Ctsb | cathepsin B | ENSMUSG00000021939 | 0.02 | 0.74 |
| Eif2ak3 | eukaryotic translation initiation factor 2 alpha kinase 3 | ENSMUSG00000031668 | 0.02 | 0.87 |
| Capn1 | calpain 1 | ENSMUSG00000024942 | 0.02 | 0.59 |
| Ctsz | cathepsin Z | ENSMUSG00000016256 | 0.02 | 0.62 |
| Pidd1 | p53 induced death domain protein 1 | ENSMUSG00000025507 | 0.02 | -0.75 |
| Ripk1 | receptor (TNFRSF)-interacting serine-threonine kinase 1 | ENSMUSG00000021408 | 0.54 | 0.03 |
| Lmnb2 | lamin B2 | ENSMUSG00000062075 | -0.50 | 0.04 |
| Itpr3 | inositol 1,4,5-triphosphate receptor 3 | ENSMUSG00000042644 | 0.56 | 0.04 |
| Tnfrsf10b | tumor necrosis factor receptor superfamily, member 10b | ENSMUSG00000022074 | 0.59 | 0.05 |

**Supplementary Table 5 continue.** Expression of genes in apoptosis pathway

**Supplementary Table 6.** Expression of genes in ferroptosis pathway

| **Gene** | **Description** | **ENSEMBL ID** | **FDR p-value** | **Log2 Fold Change** |
| --- | --- | --- | --- | --- |
| Slc11a2 | solute carrier family 11 (proton-coupled divalent metal ion transporters), member 2 | ENSMUSG00000023030 | 1.83E-29 | 3.40 |
| Slc40a1 | solute carrier family 40 (iron-regulated transporter), member 1 | ENSMUSG00000025993 | 3.94E-22 | 2.65 |
| Slc39a8 | solute carrier family 39 (metal ion transporter), member 8 | ENSMUSG00000053897 | 8.07E-20 | -2.55 |
| Gclc | glutamate-cysteine ligase, catalytic subunit | ENSMUSG00000032350 | 9.00E-15 | 2.14 |
| Ncoa4 | nuclear receptor coactivator 4 | ENSMUSG00000056234 | 3.86E-14 | 2.25 |
| Slc39a14 | solute carrier family 39 (zinc transporter), member 14 | ENSMUSG00000022094 | 5.36E-13 | 1.67 |
| Acsl3 | acyl-CoA synthetase long-chain family member 3 | ENSMUSG00000032883 | 2.18E-10 | 1.87 |
| Gclm | glutamate-cysteine ligase, modifier subunit | ENSMUSG00000028124 | 7.39E-08 | 1.31 |
| Steap3 | STEAP family member 3 | ENSMUSG00000026389 | 9.86E-07 | -2.58 |
| Hmox1 | heme oxygenase 1 | ENSMUSG00000005413 | 3.67E-06 | 1.49 |
| Sat1 | spermidine/spermine N1-acetyl transferase 1 | ENSMUSG00000025283 | 4.07E-06 | 1.24 |
| Trp53 | transformation related protein 53 | ENSMUSG00000059552 | 4.15E-06 | -1.08 |
| Fth1 | ferritin heavy polypeptide 1 | ENSMUSG00000024661 | 7.52E-06 | 1.25 |
| Ftl1 | ferritin light polypeptide 1 | ENSMUSG00000050708 | 2.68E-05 | 1.12 |
| Lpcat3 | lysophosphatidylcholine acyltransferase 3 | ENSMUSG00000004270 | 2.99E-05 | 1.19 |
| Gss | glutathione synthetase | ENSMUSG00000027610 | 6.05E-05 | 1.00 |
| Map1lc3a | microtubule-associated protein 1 light chain 3 alpha | ENSMUSG00000027602 | 8.62E-05 | 1.27 |
| Gpx4 | glutathione peroxidase 4 | ENSMUSG00000075706 | 3.76E-04 | 0.91 |
| Trf | transferrin | ENSMUSG00000032554 | 4.78E-04 | -3.11 |
| Slc7a11 | solute carrier family 7 | ENSMUSG00000027737 | 1.48E-03 | 0.95 |
| Prnp | prion protein | ENSMUSG00000079037 | 4.41E-03 | 1.89 |
| Acsl5 | acyl-CoA synthetase long-chain family member 5 | ENSMUSG00000024981 | 0.02 | 0.73 |

**Supplementary Table 7.** Expression of genes in mitophagy pathway

| **Gene** | **Description** | **ENSEMBL ID** | **FDR p-value** | **Log2 Fold Change** |
| --- | --- | --- | --- | --- |
| Optn | optineurin | ENSMUSG00000026672 | 2.33E-13 | 1.69 |
| Atg9b | autophagy related 9B | ENSMUSG00000038295 | 4.50E-12 | 2.24 |
| Jun | jun proto-oncogene | ENSMUSG00000052684 | 2.27E-08 | 1.44 |
| E2f1 | E2F transcription factor 1 | ENSMUSG00000027490 | 5.58E-08 | -1.63 |
| Rras | related RAS viral (r-ras) oncogene | ENSMUSG00000038387 | 6.09E-08 | 1.22 |
| Ubc | ubiquitin C | ENSMUSG00000008348 | 1.06E-07 | 1.37 |
| Bcl2l1 | BCL2-like 1 | ENSMUSG00000007659 | 1.09E-06 | 1.09 |
| Rabgef1 | RAB guanine nucleotide exchange factor (GEF) 1 | ENSMUSG00000025340 | 1.34E-06 | 1.10 |
| Trp53 | transformation related protein 53 | ENSMUSG00000059552 | 4.15E-06 | -1.08 |
| Csnk2a2 | casein kinase 2, alpha prime polypeptide | ENSMUSG00000046707 | 5.29E-05 | 1.13 |
| Smurf1 | SMAD specific E3 ubiquitin protein ligase 1 | ENSMUSG00000038780 | 8.07E-05 | 0.95 |
| Map1lc3a | microtubule-associated protein 1 light chain 3 alpha | ENSMUSG00000027602 | 8.62E-05 | 1.27 |
| Mfn1 | mitofusin 1 | ENSMUSG00000027668 | 2.97E-04 | 0.93 |
| Pgam5 | phosphoglycerate mutase family member 5 | ENSMUSG00000029500 | 3.55E-04 | -0.88 |
| Gabarap | gamma-aminobutyric acid receptor associated protein | ENSMUSG00000018567 | 1.29E-03 | 0.79 |
| Atg9a | autophagy related 9A | ENSMUSG00000033124 | 4.35E-03 | 0.76 |

**Supplementary Table 7 continue.** Expression of genes in mitophagy pathway

| **Gene** | **Description** | **ENSEMBL ID** | **FDR p-value** | **Log2 Fold Change** |
| --- | --- | --- | --- | --- |
| Tax1bp1 | Tax1 (human T cell leukemia virus type I) binding protein 1 | ENSMUSG00000004535 | 4.94E-03 | 0.74 |
| Pink1 | PTEN induced putative kinase 1 | ENSMUSG00000028756 | 8.29E-03 | 0.64 |
| Foxo3 | forkhead box O3 | ENSMUSG00000048756 | 8.30E-03 | 0.63 |
| Gabarapl2 | GABA type A receptor associated protein like 2 | ENSMUSG00000031950 | 8.48E-03 | 0.62 |
| Mtx3 | metaxin 3 | ENSMUSG00000021704 | 9.29E-03 | -0.74 |
| Mapk8 | mitogen-activated protein kinase 8 | ENSMUSG00000021936 | 9.93E-03 | 0.63 |
| Mfn2 | mitofusin 2 | ENSMUSG00000029020 | 0.01 | 0.62 |
| Eif2ak3 | eukaryotic translation initiation factor 2 alpha kinase 3 | ENSMUSG00000031668 | 0.02 | 0.87 |
| Bnip3l | BCL2/adenovirus E1B interacting protein 3-like | ENSMUSG00000022051 | 0.02 | 0.61 |
| Nbr1 | NBR1, autophagy cargo receptor | ENSMUSG00000017119 | 0.02 | 0.58 |
| Fundc1 | FUN14 domain containing 1 | ENSMUSG00000025040 | 0.02 | -0.71 |
| Tbk1 | TANK-binding kinase 1 | ENSMUSG00000020115 | 0.03 | 0.52 |
| Tbc1d15 | TBC1 domain family, member 15 | ENSMUSG00000020130 | 0.03 | 0.54 |
| Becn1 | beclin 1, autophagy related | ENSMUSG00000035086 | 0.03 | 0.59 |
| Siah1b | siah E3 ubiquitin protein ligase 1B | ENSMUSG00000040749 | 0.04 | -1.00 |
| Prkn | parkin RBR E3 ubiquitin protein ligase | ENSMUSG00000023826 | 0.04 | -5.80 |
| Dram2 | DNA-damage regulated autophagy modulator 2 | ENSMUSG00000027900 | 6.05E-07 | 2.16 |
